# Dynamic and regulated TAF gene expression during mouse embryonic germ cell development

**DOI:** 10.1371/journal.pgen.1008515

**Published:** 2020-01-08

**Authors:** Megan A. Gura, Maria M. Mikedis, Kimberly A. Seymour, Dirk G. de Rooij, David C. Page, Richard N. Freiman

**Affiliations:** 1 Brown University, MCB Graduate Program and Department of Molecular Biology, Cell Biology and Biochemistry, Providence, RI, United States of America; 2 Whitehead Institute, Cambridge, MA, United States of America; 3 Department of Biology, Massachusetts Institute of Technology, Cambridge, MA, United States of America; 4 Howard Hughes Medical Institute, Whitehead Institute, Cambridge, MA, United States of America; Cornell University, UNITED STATES

## Abstract

Germ cells undergo many developmental transitions before ultimately becoming either eggs or sperm, and during embryonic development these transitions include epigenetic reprogramming, quiescence, and meiosis. To begin understanding the transcriptional regulation underlying these complex processes, we examined the spatial and temporal expression of TAF4b, a variant TFIID subunit required for fertility, during embryonic germ cell development. By analyzing published datasets and using our own experimental system to validate these expression studies, we determined that both *Taf4b* mRNA and protein are highly germ cell-enriched and that *Taf4b* mRNA levels dramatically increase from embryonic day 12.5–18.5. Surprisingly, additional mRNAs encoding other TFIID subunits are coordinately upregulated through this time course, including *Taf7l* and *Taf9b*. The expression of several of these germ cell-enriched TFIID genes is dependent upon *Dazl* and/or *Stra8*, known regulators of germ cell development and meiosis. Together, these data suggest that germ cells employ a highly specialized and dynamic form of TFIID to drive the transcriptional programs that underlie mammalian germ cell development.

## Author summary

Assisted reproductive therapy and fertility preservation are increasingly used to improve human reproduction across the world, yet there are still many unanswered questions regarding what factors govern the development of eggs and sperm and how these factors work together. We previously identified a subunit of the general transcription factor TFIID, TAF4b, that is essential for fertility. However, many basic characteristics of how *Taf4b* and its associated TFIID family members contribute to the formation of healthy sperm and eggs in mice and humans remain unknown. In this study, we find that mouse *Taf4b* and several closely related TFIID subunits become highly abundant during mouse embryonic gonad development, specifically in the cells that ultimately become eggs and sperm. Here, we analyzed data from public repositories and isolated these developing cells to examine their gene expression patterns throughout embryonic development. Together these data suggest that the dynamic expression of *Taf4b* and other TFIID family members are dependent on the well-established reproductive cell regulators *Dazl* and *Stra8*. This understanding of *Taf4b* gene expression and regulation in mouse reproductive cell development is likely conserved during development of human cells and offers novel insights into the interconnectedness of the factors that govern the formation of healthy eggs and sperm.

## Introduction

Healthy development and maintenance of germ cells is essential for the continuation of all sexually reproducing species. In the United States, approximately 10% of individuals face fertility issues, and worldwide, at least 1% of females experience primary ovarian insufficiency (POI), which is associated with infertility [1,2]. However, in most instances of POI and infertility, the underlying molecular causes are unknown [3]. Work from our laboratory has shown that a *TBP-Associated Factor 4b* (*Taf4b*)-deficient mouse model recapitulates many aspects of POI, including a premature depletion of the ovarian reserve and female infertility [4,5]. *Taf4b*-deficiency in the mouse also leads to progressive male infertility in early adulthood that is associated with exhaustion of their adult spermatogonial stem cell (SSC) pool [6]. Since human studies implicate TAF4B as important for fertility and oocyte quality [7–9], our efforts to understand the molecular mechanisms underlying the expression and function of TAF4b in mouse germ cell development may contribute to our increased understanding of human fertility and infertility.

TAF4b is a gonad-enriched subunit of the basal transcription factor TFIID, which is a complex of TATA-binding protein (TBP) and 13–14 TBP-associated factors (TAFs) [5,10]. TFIID, as part of a larger ensemble of the basal transcription machinery, recruits RNA Polymerase II (RNAPII) to the core promoters of genes. However, unlike its paralog *Taf4a*, which is expressed ubiquitously, *Taf4b* is more highly expressed in the mouse ovary and testis compared to other tissues [5]. Most recently, we demonstrated that TAF4b is a critical regulator of female meiosis I in the mouse [11]. *Taf4b* mRNA expression is also highly correlated with the expression of many important germline genes during human fetal ovary development, such as *Deleted in Azoospermia-like* (*Dazl*), which encodes a germ cell-specific RNA-binding protein that promotes translation and/or prevents the degradation of its target mRNAs [11–13]. In female mice as early as E13.5, when germ cells initiate meiosis, *Taf4b*-deficiency results in reduced expression of meiotic transcripts, including *Stimulated by Retinoic Acid 8* (*Stra8)*, which is a master regulator of meiosis [14]. This reduced meiotic gene expression is followed by defective meiotic progression, and excessive perinatal oocyte loss [11]. *Stra8* mRNA has also been found to be greatly reduced in *Taf4b*-deficient male newborn testes compared to wild-type and *Taf4b*-heterozygous testes, indicating that this disruption of meiosis is likely shared between female and male *Taf4b*-deficient mice [15].

We previously detected TAF4b occupancy at the proximal promoters of *Dazl* and *Stra8* in E18.5 embryonic ovaries, which suggests that TAF4b directly regulates these genes [11]. *Stra8* was recently identified as a major transcriptional regulator of meiotic initiation [14]. However, it was postulated that *Stra8* alone is not sufficient to induce meiosis, and other epigenetic and transcriptional regulators likely work together with *Stra8* to orchestrate meiotic initiation. It is unknown what transcriptional program coordinates with *Stra8* in germ cells to produce gametes.

While female germ cells initiate meiosis at E13.5, male germ cells remain mitotically active until ~E16.5 [16]. During this time, *Taf4b*-deficient male mice display deficient embryonic gonocyte numbers, as well as defective postnatal germ cell proliferation and development [6]. Therefore, during early postnatal development, a paucity of germ cells leads to a narrow window of fertility that may result from an inability to establish a healthy embryonic germ cell and/or postnatal SSC pool. One common theme in both adult females and males is that their *Taf4b*-dependent fertility deficits can be traced back to defects in embryonic germ cell development.

Despite the knowledge that *Taf4b* function is essential for embryonic germ cell development in both sexes, the precise expression profile and mechanism of action for this fertility transcription factor remains unknown. Thus, it is uncertain whether *Taf4b* uses similar molecular mechanisms in female and male germ cells. This gap in knowledge makes understanding the role of *Taf4b* in embryonic gametogenesis more challenging. To develop a better understanding of *Taf4b*, we implemented complementary computational and experimental tools to assess the sex-specific timing and localization of *Taf4b* expression in the embryonic mouse gonad. We found that *Taf4b* mRNA and protein are highly enriched in the germ cells of the embryonic gonad and that strikingly similar expression patterns were observed for other TFIID subunits, such as *Taf7l*, which is essential for male germ cell maturation and fertility [17,18]. In addition, we demonstrate that *Taf4b* is the only component of the TFIID and SAGA complexes that is directly regulated by both DAZL and STRA8 during meiosis. Based upon these data, *Taf4b* functions in an interdependent gene regulatory network with DAZL and STRA8 during meiosis.

## Results

### *Taf4b* is significantly enriched and dynamically expressed in the germ cells of the embryonic ovary and testis

**Fig 1:**
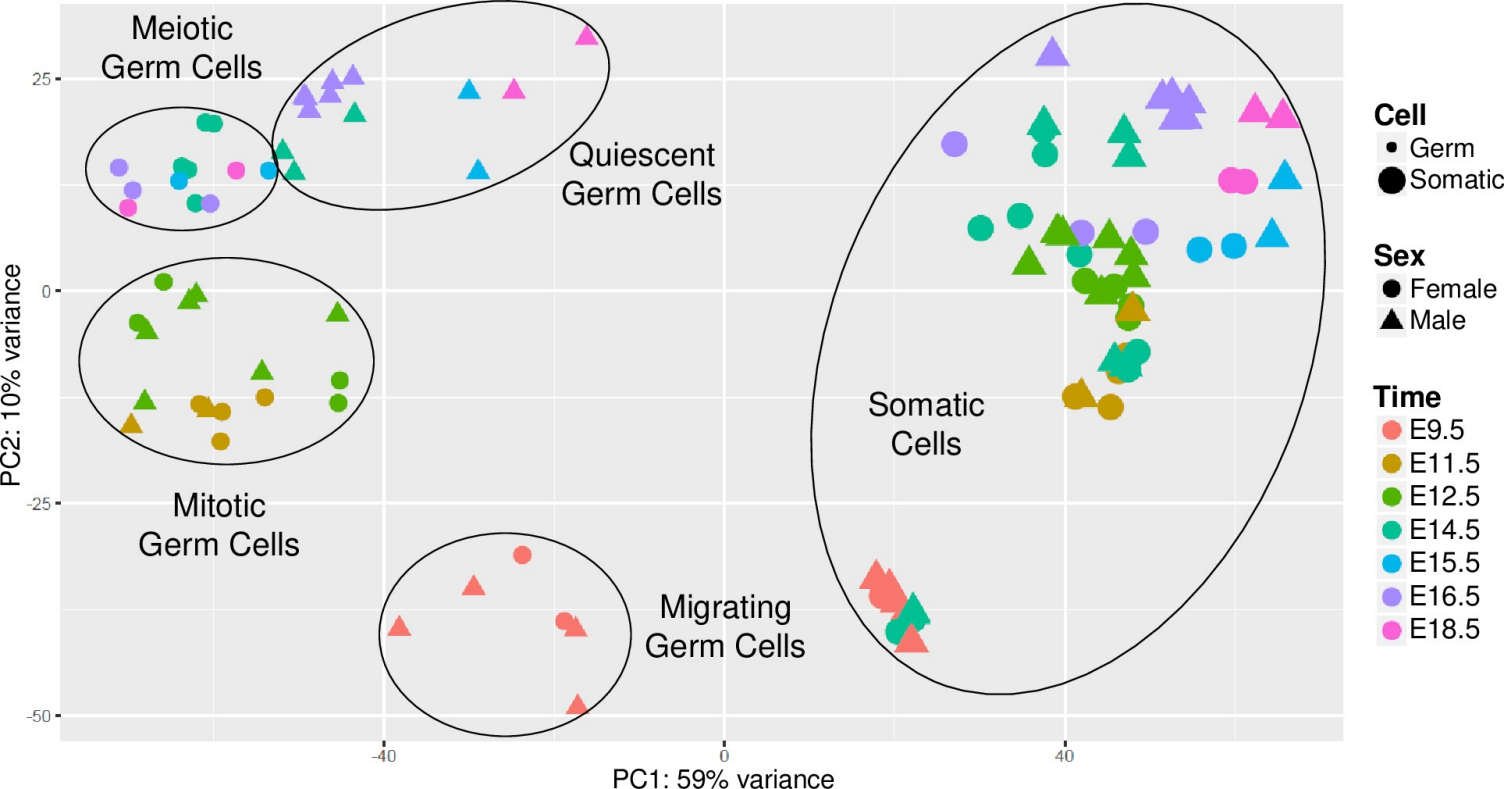
A comprehensive RNA-seq time course dataset from Sangrithi et al. in the cells of the embryonic gonad [19]. PCA plot of the reprocessed mouse RNA-seq data. Somatic and germ cell lineages (dot size) are separate on the plot, germ cells group together depending on stage of development (dot color) and later by sex (dot shape).

To evaluate *Taf4b* expression in the embryonic gonad, we reprocessed an RNA-sequencing (RNA-seq) dataset that used Oct4-EGFP mice covering 7 developmental time points, which range from embryonic day 9.5 (E9.5) to E18.5, wherein each time point had appropriate female and male replicates [19,20]. This sex-specific time course was further broken down into germ cell (GFP^+^) and gonadal somatic (GFP^-^) cells by performing fluorescence activated cell sorting (FACS). Principal component analysis (PCA) (**Fig 1**) indicates distinct grouping of germ cells and somatic cells on opposite sides of the PC1 dimension and further grouping of germ cells based on their developmental milestones.

**Fig 2:**
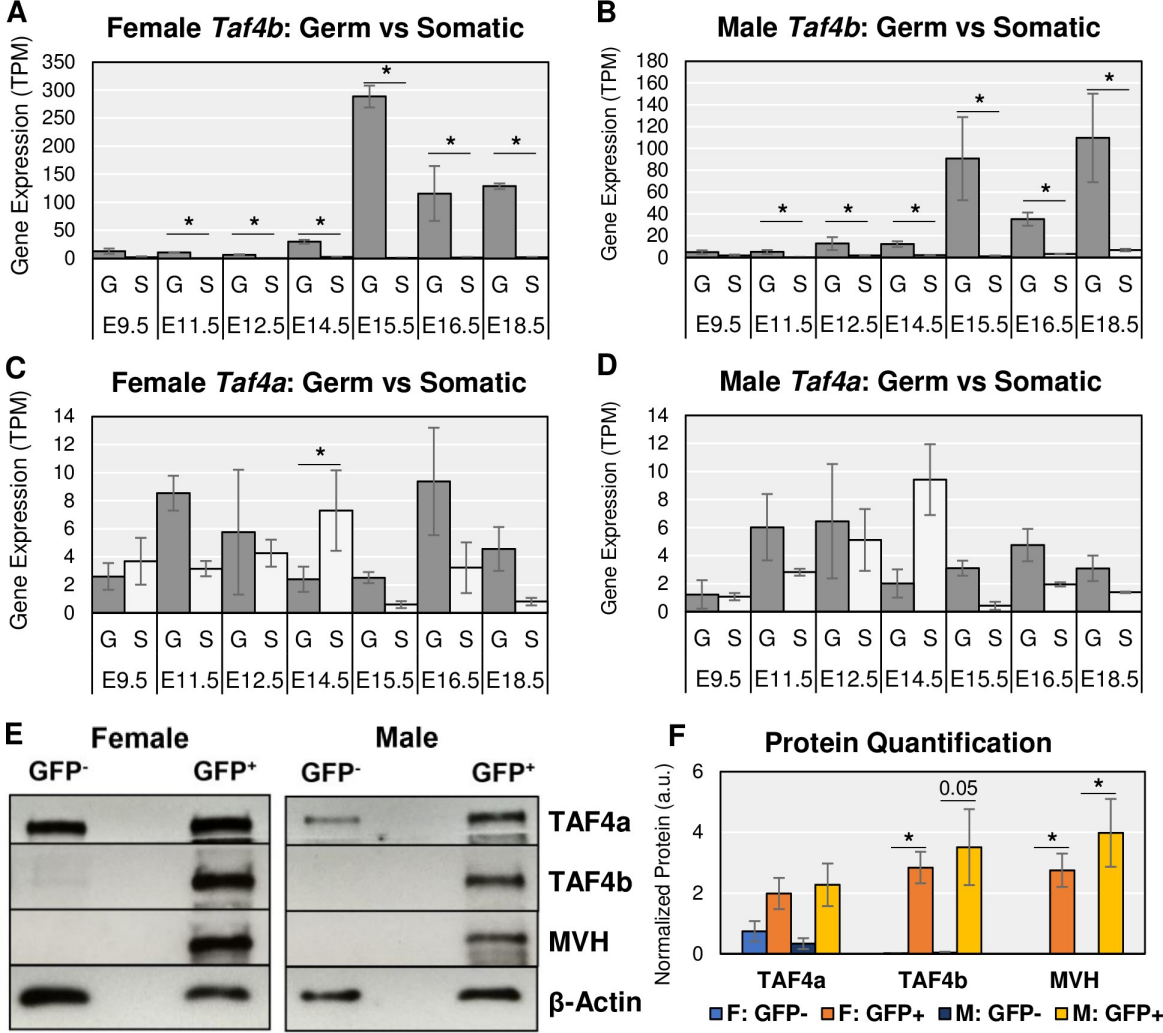
*Taf4a* vs *Taf4b* enrichment in the germ cells of the mouse embryonic gonad. Gene mRNA expression levels of *Taf4b* (A-B) and *Taf4a* (C-D) in female and male embryonic germ cells (“G”) and somatic cells (“S”) from E9.5 to E18.5 indicate that *Taf4b* is significantly (* = log2FC > |0.25|, p-adj. < 0.05) and consistently enriched in germ cells from E11.5 to E18.5 in comparison to somatic cells. Error bars indicate ± standard error of the mean (SEM). (E) Western blot protein signal of TAF4a and TAF4b in cells sorted from E13.5 Oct4-EGFP gonads. Mouse Vasa Homology (MVH) is a germ cell marker, which is only detected in the GFP^+^ lanes of the female and male samples, indicating that the GFP^-^ lane contains only somatic cells. TAF4a protein signal is detected in all lanes (both germ and somatic cell types), whereas TAF4b is only detected in the GFP^+^ lane (germ cells). β-Actin is a protein loading control. Two female mice and two male mice were used to obtain roughly 35,000 pooled cells for each lane. (F) Western blot experiment performed in 3 replicates was quantified for relative protein signal. TAF4b protein levels between GFP^+^ and GFP^-^ cells in female E13.5 ovary are significantly (* = p < 0.05, unpaired t-test) different. MVH is significantly different for both females and males. Error bars indicate ± SEM.

We first examined in which embryonic gonadal cell type *Taf4b* mRNA is expressed since the embryonic ovary and testis are heterogeneous tissues composed of germ and somatic cells. In both tissues from E11.5 to E18.5, *Taf4b* mRNA is significantly and consistently enriched in the GFP^+^ germ cells (**Fig 2A and 2B**, log_2_FC > |0.25|, p-adj. < 0.05, **S1 Table**). *Taf4b* expression, shown in transcripts per million (TPM, **S2 Table**), remains relatively low in all gonadal somatic cells throughout the time course. The slight increase in *Taf4b* mRNA in male somatic samples at E18.5 (**Fig 2B**) is not significant. Thus, *Taf4b* mRNA expression is preferentially expressed in the germ cells of the embryonic gonad. We hypothesize that *Taf4b* plays a specialized role in germ cell development and fertility in contrast to its more ubiquitously expressed paralog *Taf4a*. We thus compared *Taf4a* mRNA to *Taf4b* expression levels in this dataset (**Fig 2C and 2D**). In contrast to *Taf4b*, *Taf4a* mRNA expression was in general not significantly different between germ cell and somatic cell types. Furthermore, *Taf4a* mRNA was detected at lower TPMs over the entire time course, which indicates that *Taf4a* mRNA is reduced compared to *Taf4b* mRNA.

To test if the enrichment of *Taf4b* mRNA within germ cells is reflected at the protein level, we performed western blots on whole cell protein extracts of E13.5 germ and somatic cells derived from a similar Oct4-EGFP mouse line. At E13.5, the signal for mouse vasa homolog (MVH), a germ cell-specific marker, is only found in the GFP^+^ protein samples of female and male cells (**Fig 2E**). This marker expression indicates that we successfully separated the germ cells from the somatic cells. Based on quantification of western blot signal from 3 biological replicates, TAF4b protein expression is predominantly detected in the GFP^+^ germ cells from both female and male samples, whereas TAF4a is expressed in both GFP^+^ germ cells and GFP^-^ somatic cells (**Fig 2E and 2F**, **S1A–S1D Fig**). Even at higher GFP^-^ somatic cell numbers, a TAF4b protein signal is barely detectable (**S1E Fig**), indicating that TAF4b protein is strongly enriched in the germ cells of E13.5 mouse gonads and suggesting that the expression of both *Taf4b* mRNA and protein during embryonic reproductive development is primarily within the germ cells.

**Fig 3:**
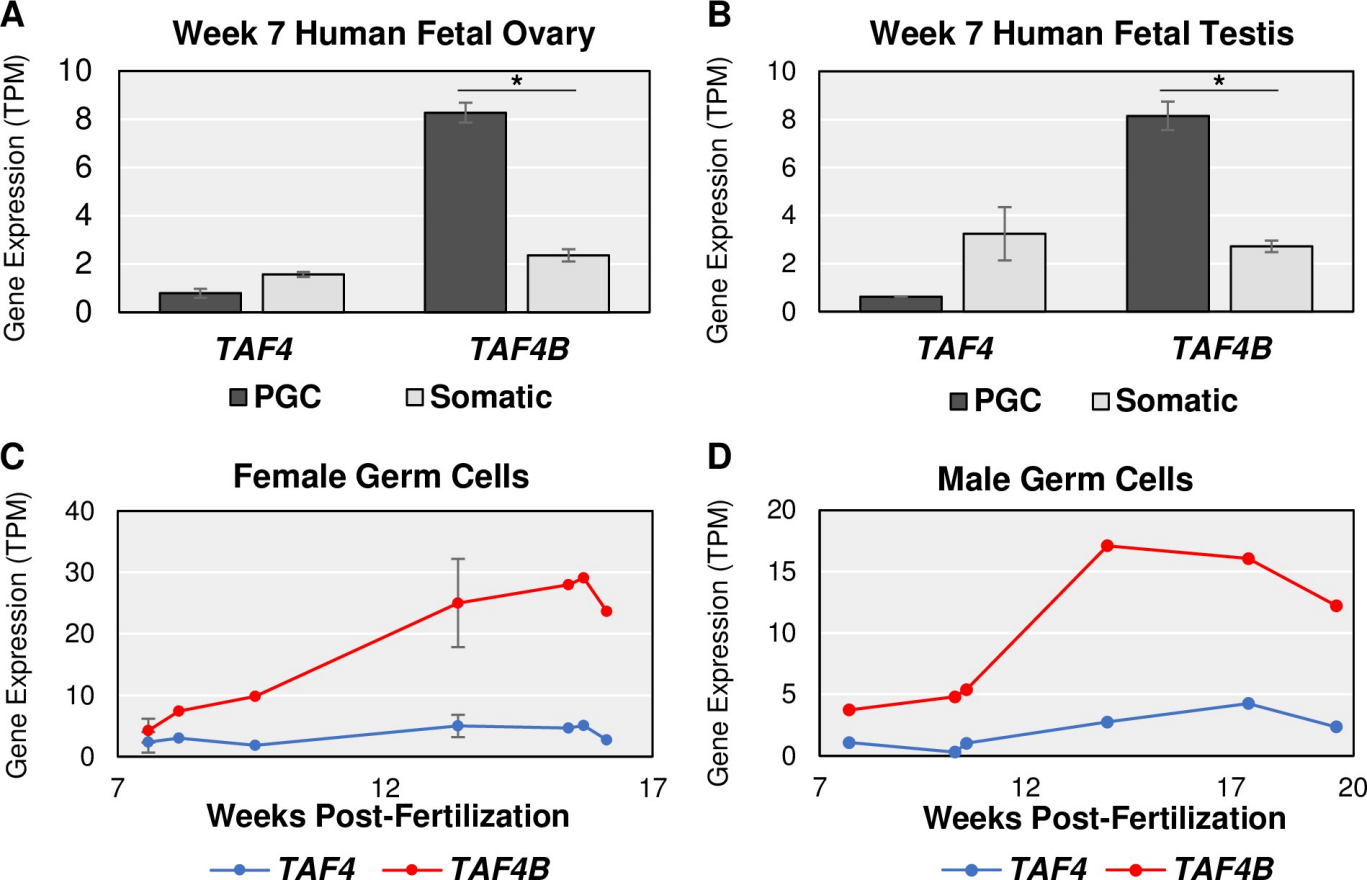
Human embryonic data resembles *Taf4a* and *Taf4b* characteristics in mouse. (A-B) At 7 weeks post-fertilization, female gonadal cells sorted for alkaline phosphatase-positive, CD117-positive germ cells (PGC) and male gonadal cells sorted for cKit-positive, TNAP-positive PGCs have significantly (* = log_2_FC > |0.25|, p-adj. < 0.05) greater *TAF4B* mRNA in comparison to the gonadal somatic cells (Somatic) in both females and males. *TAF4* mRNA levels are not significantly different in the two cell populations in both sexes, like the mouse. (C-D) Using cKit-positive germ cells to perform FACS, from ~8 weeks post-fertilization to ~16 weeks post-fertilization in females and to ~20 weeks post-fertilization in males, human *TAF4B* mRNA expression increases more so than *TAF4*. Also, *TAF4B* TPMs is greater in the female human germ cells than the male germ cells, making the expression patterns of *TAF4B* over time very similar between mice and humans. Error bars indicate ± SEM.

Given its expression in germ cells, we more closely examined the mRNA expression of *Taf4b* versus *Taf4a* in the E9.5-E18.5 time course. *Taf4b* dramatically increases at E15.5 in both female and male germ cells while *Taf4a* expression is relatively constant and much lower (**Fig 2A–2D**, dark gray bars). However, the female expression of *Taf4b* reaches greater TPM than the males, as indicated by the differences in the scale of the y-axes. Similar *Taf4b* and *Taf4a* mRNA expression profiles can be reliably found in independent RNA-seq datasets that isolate germ cells from the embryonic gonad (**S2 Fig**). These data suggest that as germ cells progress through development, and enter meiosis in female germ cells, *Taf4b* mRNA and protein are available to play a role in these processes. In addition, when the human embryonic gonad is sorted into germ and somatic cell populations, *TAF4B* mRNA expression is similarly germ cell-enriched (**Fig 3A and 3B**), and increases over comparable time points in the available human datasets (**Fig 3C and 3D**, **S3–S5 Tables**), indicating that mouse and human TAF4B may play analogous roles during embryonic germ cell development [21–23].

While we identified many differentially expressed genes in pairwise comparisons of germ versus somatic cell gene expression across time (**S1 Table**), we used the program ImpluseDE2 [24] for time course differential expression analysis in female and male germ cells. ImpulseDE2 determined that *Taf4b*, but not *Taf4a* mRNA expression was significant (p-adj. < 0.05) across both female and male germ cell timelines, referred to as “significantly dynamic” (**S6 Table**). Together these data suggest that *Taf4b* expression is nearly exclusive to the germ cells of the embryonic gonad and in the female this corresponds to our time period of interest, when meiosis and primordial follicle development have initiated.

### Many TFIID subunits are dynamically expressed in embryonic germ cells

**Fig 4:**
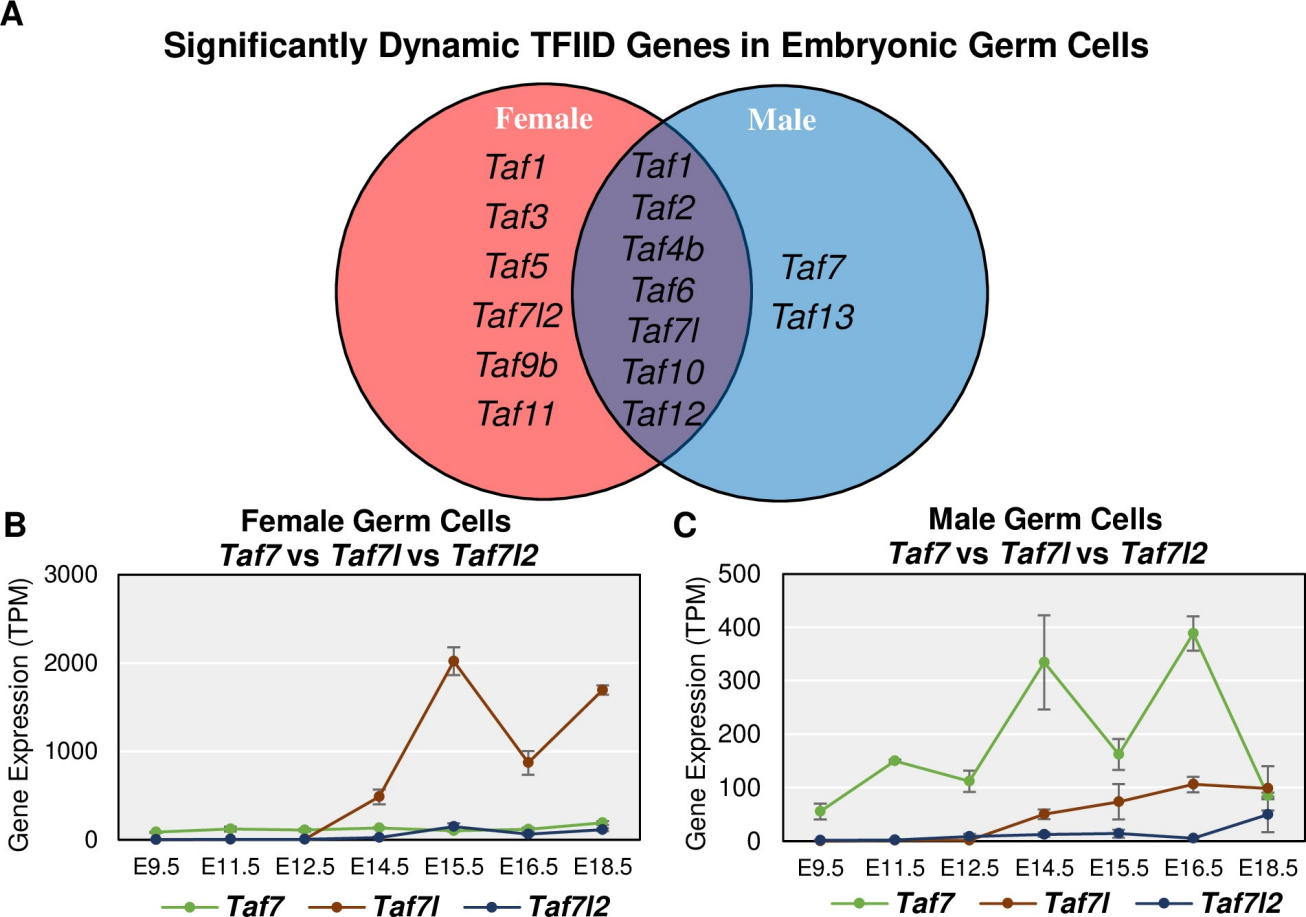
Many TFIID components changing during embryonic germ cell development. (A) Venn diagram of TFIID subunits identified by ImpulseDE2 as significantly dynamic in the female and/or male germ cell time course data. (B-C) Gene expression levels for *Taf7*, *Taf7l*, and *Taf7l2* in female and male germ cells, respectively. *Taf7l* is most highly expressed in female germ cells, but *Taf7* is expressed more in male germ cells. Error bars indicate ± SEM.

Using these ImpulseDE2 data, we asked whether other subunits of the TFIID complex were co-regulated with *Taf4b*. To our surprise, many other TFIID subunits were also found to be significantly dynamic in the female timeline, male timeline, or both (**Fig 4A**). One especially notable TAF variant that was found to be significantly dynamic in both female and male embryonic germ cells was *Taf7l*. *Taf7l* is X-linked and known to be essential for male fertility [17,18]. Interestingly, *Taf7l* is a paralog of *Taf7* and has an autosomal retrotransposed copy called *Taf7l2* (previously known as “4933416C03Rik” [14]). ImpulseDE2 identified *Taf7l2* and *Taf7* as significantly dynamic in the female and male germ cells, respectively. In female germ cells, *Taf7l* mRNA expression drastically increases starting at E14.5 and remains high (**Fig 4B**). *Taf7l2* appears to mimic the female gene expression trajectory of *Taf7l* albeit at a lower TPM level (S3**A Fig**). In male germ cells, *Taf7* is the most abundant of the three, with *Taf7l* increasing in expression at E14.5, like its female germ cell expression (**Fig 4C**). When looking more closely at only *Taf7l* and *Taf7l2*, we see that *Taf7l* begins to increase in expression at E14.5, like in females, but *Taf7l2* appears to increase in expression around E18.5 and does not mimic the male trajectory of *Taf7l* (**S3B Fig**). These data suggest that both embryonic female and male germ cells share *Taf7l* expression but express *Taf7l2* and *Taf7* in female- and male-specific ways, respectively.

Furthermore, the germ cell enrichment or specificity of *Taf7l*, *Taf7l2*, and *Taf7* depends on the sex of the germ cells. In females, *Taf7l* and *Taf7l2* are significantly and consistently germ cell-specific starting at E14.5 whereas in males it is *Taf7* and *Taf7l* that are significantly and consistently germ cell-enriched (**S3C–S3H Fig**). This implicates *Taf7l* as a shared TFIID component of importance between female and male germ cells while *Taf7l2* and *Taf7* display a female or male germ cell-specific preference, respectively. However, in the available human data, *TAF7L* is not detectable in the embryonic gonad 8–20 weeks post fertilization, *TAF7* shows a non-significant preference for somatic cell expression at 7 weeks post-fertilization, and *Taf7l2* is a rodent-exclusive retrogene which altogether might indicate that this trio is less important in human prenatal germ cell development (**S3I–S3L Fig**).

Since both *Taf4b* and *Taf7l* are germ cell-enriched, and several TFIID subunits were identified as significantly dynamic by ImpulseDE2 in the mouse, we asked if any other components of TFIID are also germ cell-enriched like *Taf4b* and *Taf7l*. If individual TFIID components had significantly (log_2_FC > |0.25|, p-adj. < 0.05) higher mRNA expression in the germ cells over the somatic cell samples for at least 3 of the 7 measured time points in either sex, it was considered “germ cell-enriched”. We found that 13 additional TFIID-encoding genes were germ cell-enriched (S3**M Fig**), demonstrating that germ-cell enrichment of TFIID complex genes is not unique to *Taf4b* and *Taf7l*.

### *Taf4b*, *Taf7l* and *Taf9b* cluster with critical germ cell development and meiotic genes

**Fig 5:**
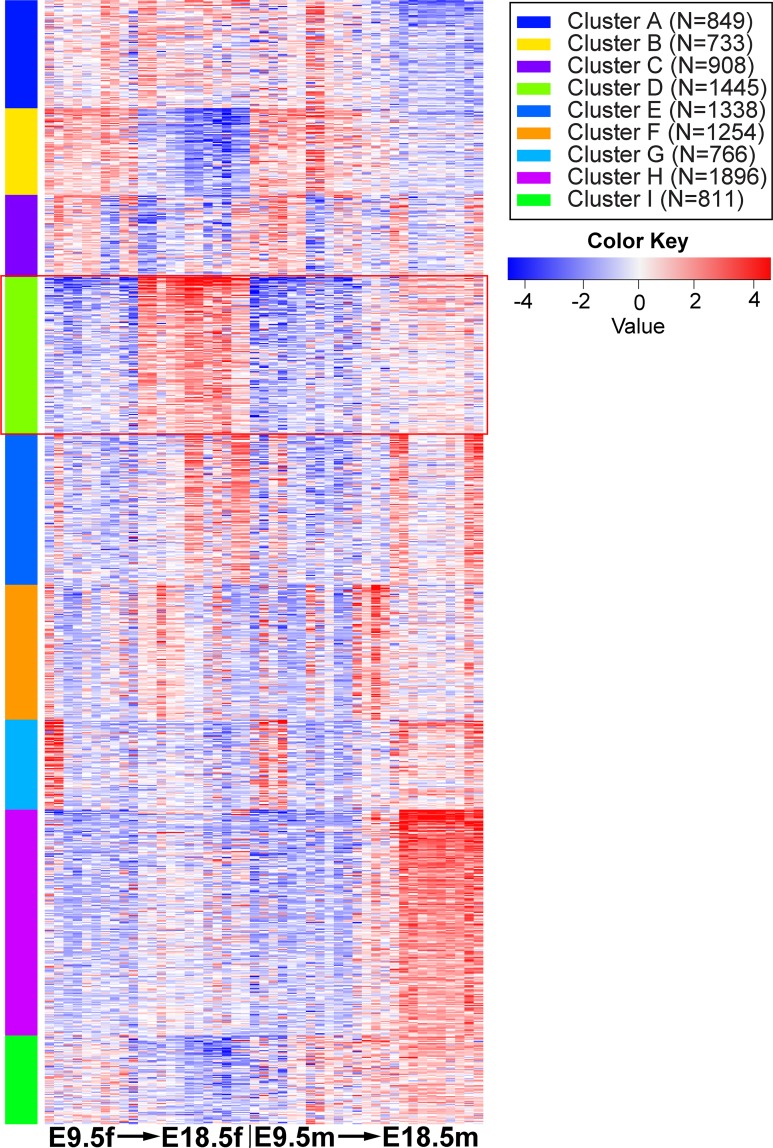
K-means clustering of female and male germ cell samples in Oct4-EGFP mouse time course. Heatmap generated based on clustering the top 10,000 most variable genes in the dataset into 9 clusters. Red box around Cluster D to identify *Taf4b*-containing group of genes.

The enrichment and dynamic expression of TFIID subunits in both female and male germ cells led us to question if these TFIID components were coordinately expressed. We performed k-means clustering on the top 10,000 most variable genes across all germ cell samples in the reprocessed mouse time course dataset (**Fig 5**). Nine clusters were chosen based on the elbow method of determining k clusters (**S4 Fig**). Using this clustering method, 11 TAFs were found in our 9 clusters (**Table 1**). Cluster D (**Fig 5**, red box) is particularly interesting because 5 TAFs (*Taf4b*, *Taf5*, *Taf7l*, *Taf7l2*, and *Taf9b*) clustered with genes highly relevant to overall germ cell development (*Dazl*, *Ddx4*, *Ybx2*) and meiosis I (*Stra8*, *Meioc*, *Ythdc2*). Furthermore, gene ontology of Cluster D indicates that there is significant enrichment of meiosis I and germ cell development genes in this group (**Table 1**, **S7 Table**).

**Table 1:** Top gene ontology category in each cluster, TFIID component found in each, and other notable genes found in cluster.

Cluster	TFIID	Top GO Biological Process Category	Adjusted p-value	Other Notable Genes
**A**	*Taf12*	Cellular macromolecule biosynthetic process	1.29E-24	*Ccne1*, *Sohlh2*
**B**		Small molecule metabolic process	1.52E-20	*Aurkc*, *Cdk1*
**C**		Nucleic acid metabolic process	1.28E-15	*Ccna2*
**D**	*Taf4b*, *Taf5*, *Taf7l*, *Taf7l2*, *Taf9b*	Meiosis I	5.54E-17	*Ddx4*, *Dazl*, *Meioc*, *Stra8*, *Ythdc2*, *Ybx2*
**E**	*Taf10*	Cellular protein modification process	3.56E-16	*Cdk2*, *Foxo3*, *Id4*
**F**		System development	8.06E-10	*Zbtb16*
**G**		Tube development	8.05E-24	*Sox9*
**H**	*Taf2*	System development	3.06E-20	*Sohlh1*, *Mael*
**I**	*Taf1*, *Taf4a*, *Taf7*	Regulation of nucleic acid-templated transcription	1.14E-06	*Dmrt1*

It is very interesting to find that *Taf4b*, a gene with known connections to meiotic genes, clusters with *Taf5*, *Taf7l*, *Taf7l2*, and *Taf9b*, which have not been previously associated with meiosis. Furthermore, all but *Taf5* are alternative TAFs, meaning that their place in the TFIID complex is not guaranteed but could instead be occupied by their counterpart. For example, both TAF4a and TAF4b are capable of replacing each other in TFIID.

### Loss of *Dazl* and *Stra8* regulation affect proper expression of many TFIID components, including *Taf4b*

To further examine the potential relevance of TFIID in germ cells and what could be connecting their expression patterns, we reprocessed three RNA-seq datasets from *Dazl*- and *Stra8*-KO female and male mice [13,14,25]. Both *Dazl* and *Stra8* are germ cell-specific regulators of gene expression that are essential for meiosis and fertility. *Dazl* encodes an RNA-binding protein that may promote translation of its target mRNAs and/or stabilize transcripts to prevent their degradation [12,13]. *Stra8* encodes a sequence-specific transcriptional activator that amplifies meiotic and cell cycle genes to initiate meiosis I [14].

**Fig 6:**
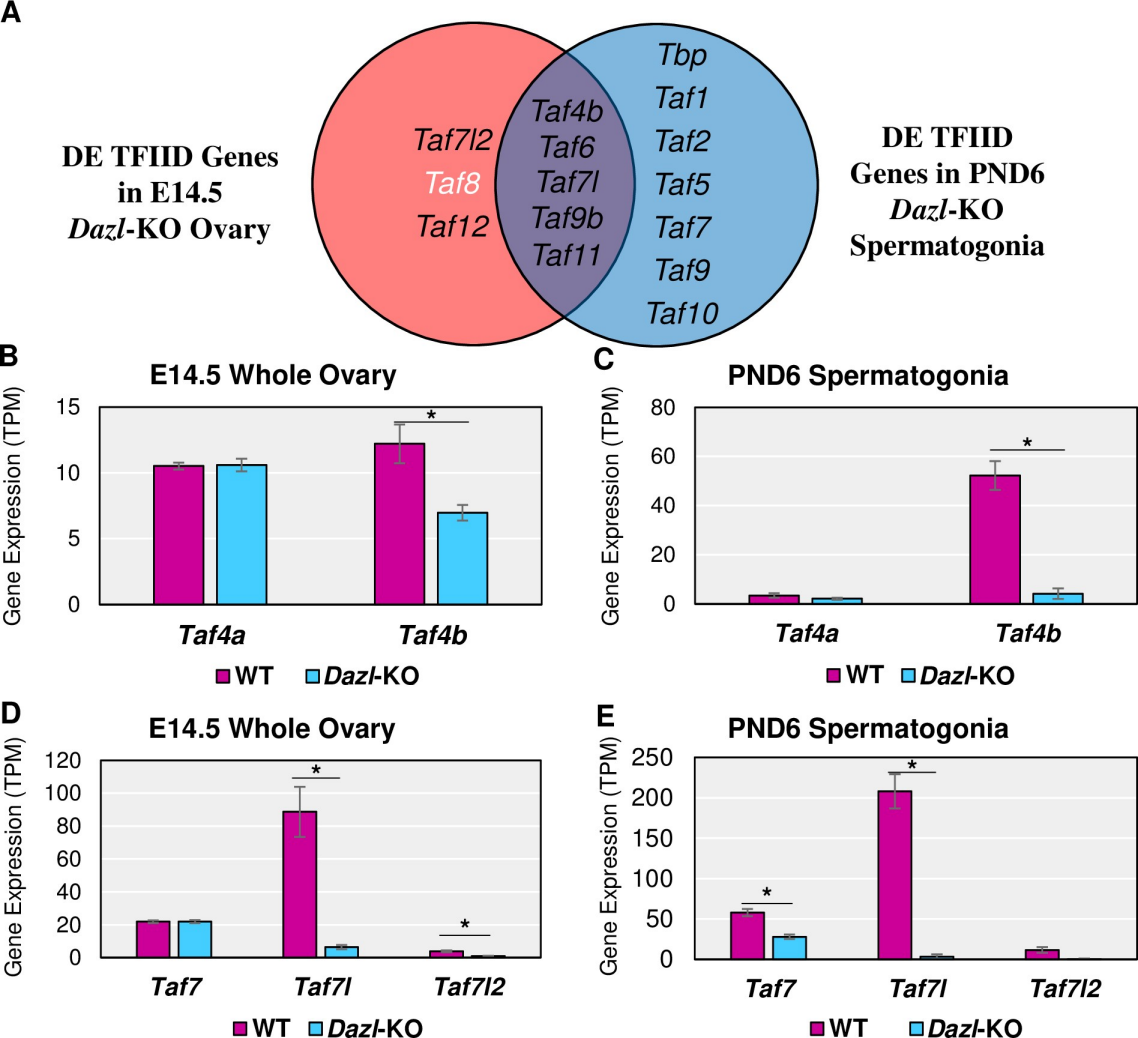
TFIID subunits in *Dazl*-KO female and male mice. (A) Venn diagram of TFIID subunits found to be significantly (log_2_FC > |0.25|, p-adj < 0.05) different between the WT and *Dazl*-KO of the E14.5 ovary and/or PND6 spermatogonia. Black text indicates decreased mRNA expression in the *Dazl*-KO and white text indicates increased mRNA expression in the *Dazl*-KO compared to WT. Gene expression levels of *Taf4a* versus *Taf4b* in female (B) and male (C) WT versus *Dazl*-KO RNA-seq experiments. Gene expression levels of *Taf7*, *Taf7l*, and *Taf7l2* in female (D) and male (E) WT versus *Dazl*-KO experiments (* = log_2_FC > |0.25|, p-adj. < 0.05). Error bars indicate ± SEM.

To determine which TAFs, if any, were deregulated when *Dazl* expression is disrupted, we reprocessed two *Dazl*-KO RNA-seq datasets. For the female *Dazl*-KO, Soh et al. performed RNA-seq on E14.5 whole ovaries with a global disruption of the *Dazl* gene (**S8 Table**). *Dazl* is expressed in a germ cell-specific manner, so noise from the somatic cells of the ovary is minimized. For the male *Dazl*-KO, Zagore et al. used the same *Dazl* mutation but combined its usage with a *Stra8-Cre*; IRG^+^ system, which expresses RFP before Cre recombination and EGFP after, so that EGFP^+^ spermatogonia could be collected via FACS at PND6 despite low germ cell numbers (**S9 Table**) [26]. Even with different female and male biological contexts in these RNA-seq datasets, we found many components of the TFIID complex to be significantly affected (log_2_FC > |0.25|, p-adj < 0.05), with *Taf4b*, *Taf7l*, and *Taf9b* significantly reduced in both sexes of the *Dazl*-KO mouse (**Fig 6A**). Closer examination of these data indicates that *Taf4b*, but not *Taf4a*, is significantly decreased in the female and male *Dazl*-KO mouse (**Fig 6B and 6C**). Additionally, *Taf7l* and *Taf7l2* are significantly decreased in the female *Dazl*-KO data while *Taf7* and *Taf7l* are significantly decreased in the male *Dazl*-KO (**Fig 6D–6E**).

**Fig 7:**
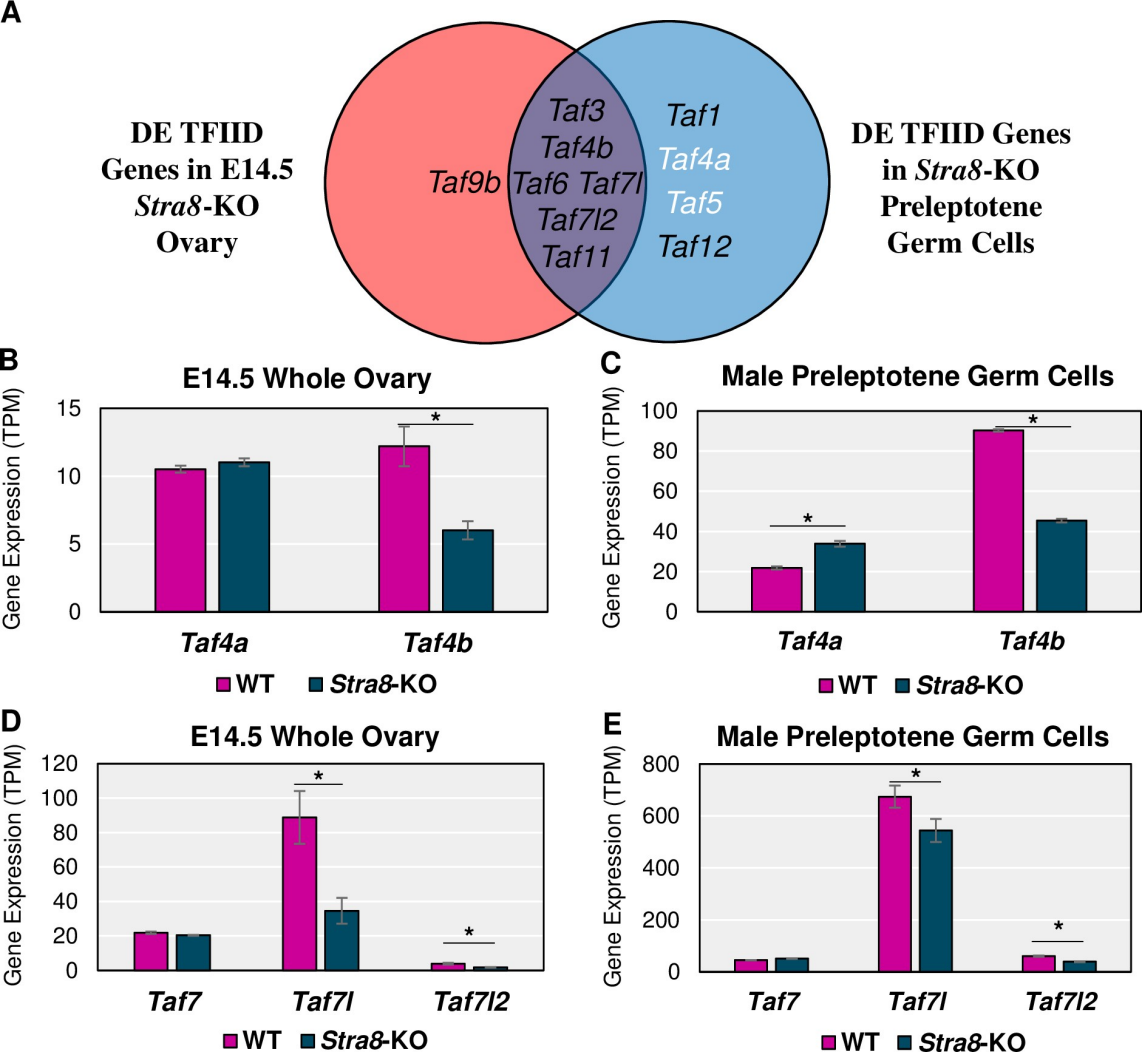
TFIID subunits in *Stra8*-KO female and male mice. (A) Venn diagram of TAFs found to be significantly (log_2_FC > |0.25|, p-adj < 0.05) different between the WT and *Stra8*-KO of the E14.5 ovary and/or male preleptotene germ cells. Black text indicates decreased mRNA expression in the *Stra8*-KO and white text indicates increased mRNA expression in the *Stra8*-KO compared to WT. Gene expression levels of *Taf4a* versus *Taf4b* in female (B) and male (C) WT versus *Stra8*-KO RNA-seq experiments. Gene expression levels of *Taf7*, *Taf7l*, and *Taf7l2* in female (D) and male (E) WT versus *Stra8*-KO experiments (* = log_2_FC > |0.25|, p-adj. < 0.05). Error bars indicate ± SEM.

To determine which TAFs, if any, might be dependent on *Stra8* expression, we reanalyzed two *Stra8*-KO RNA-seq datasets. For the female *Stra8*-KO, Soh et al. also performed RNA-seq on E14.5 whole ovaries in a global disruption in the *Stra8* gene (**S8 Table**). For the male *Stra8*-KO, Kojima et al. used the same *Stra8*-KO crossed to a *Ddx4-Cre*; *Rosa26-tdTomato* mouse line and isolated preleptotene male germ cells (**S10 Table**). Like in the *Dazl*-KO data, *Taf4b* and *Taf7l* were significantly reduced in the female and male *Stra8*-KO gonads along with many other TAFs, including *Taf9b* in the females (**Fig 7A**).

The mRNA dynamics of *Taf4a* and *Taf4b* in the *Stra8*-KO data are much like the *Dazl*-KO data, with *Taf4b* being significantly decreased in the *Stra8*-KO female and male samples (**Fig 7B and 7C**). Interestingly, *Taf4a* is significantly increased in the *Stra8*-KO preleptotene germ cells. Both *Taf7l* and *Taf7l2* are significantly decreased in the *Stra8*-KO but *Taf7* is unchanged (**Fig 7D and 7E**). These data suggest that not only do DAZL and STRA8 regulate several TFIID components not traditionally thought of as part of the germ cell development gene program, but also that *Taf4b* and *Taf7l* are consistent, active members of both the female and male germ cell-specific programs of gene expression.

### DAZL and STRA8 directly bind to many of the TFIID components improperly expressed in *Dazl*- and *Stra8*-KO mice

To inquire as to whether DAZL may directly regulate any TFIID components, we identified DAZL-bound transcripts via individual-nucleotide resolution cross-linking and immunoprecipitation (iCLIP) (S5**A Fig**) [27]. Given that we have previously shown that *Taf4b* impacts meiotic prophase I in females and males [11], we sought to understand whether *Taf4b* is regulated by DAZL in a meiotic context, focusing on the leptotene stage of meiotic prophase I. To obtain the large number of leptotene spermatocytes needed for iCLIP, we developmentally synchronized spermatogenesis by chemically regulating the levels of retinoic acid, which is required for spermatogonial differentiation [28,29]. This synchronized development allowed us to collect testes enriched for leptotene spermatocytes. The successful accumulation of leptotene spermatocytes, without contamination from other spermatocyte stages, was verified in a testis biopsy via histological analysis [30].

**Fig 8:**
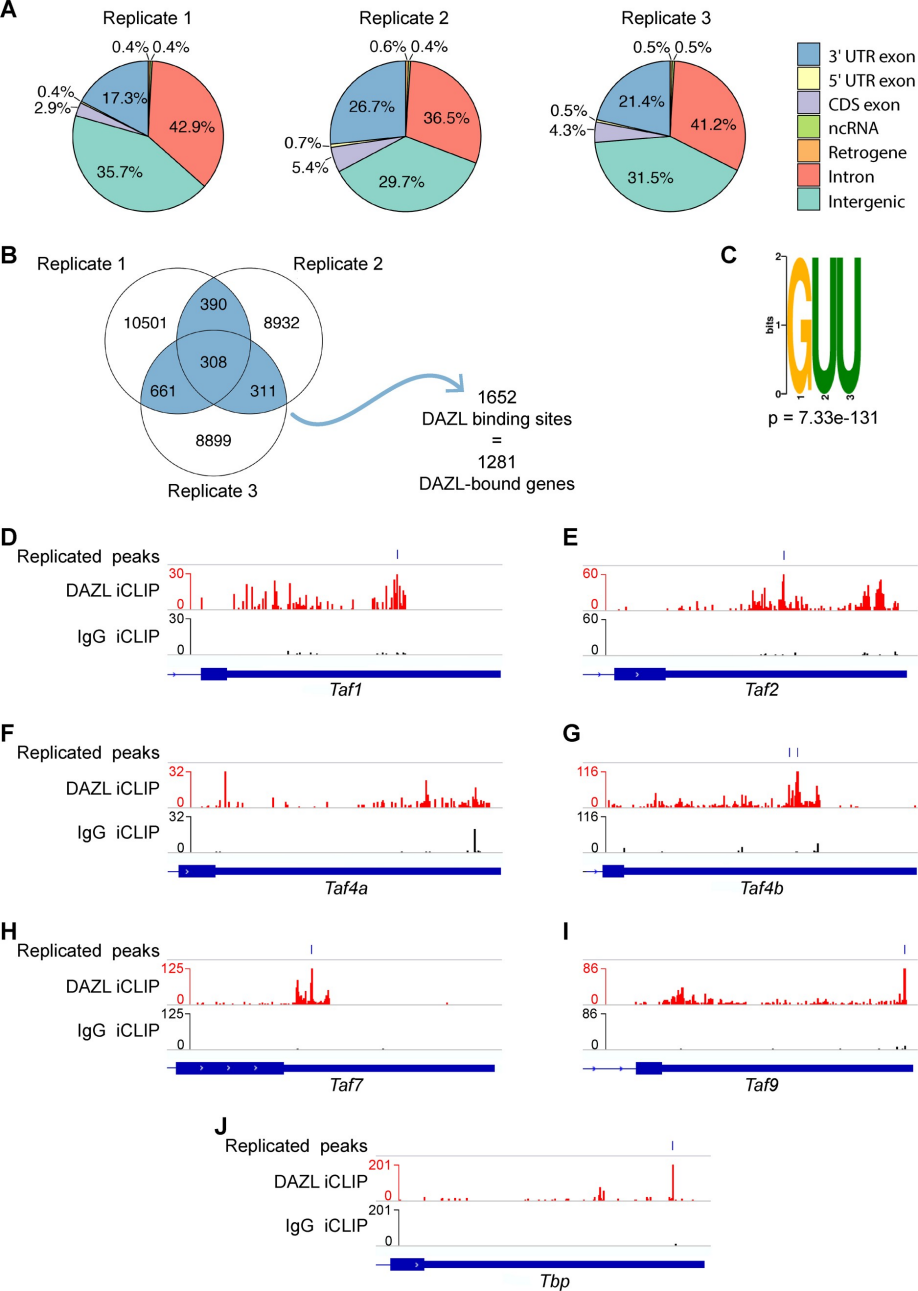
Identification of DAZL targets via iCLIP in testes synchronized for leptotene spermatocytes. (A) Genomic distribution of DAZL iCLIP peaks identified in 3 biological replicates (p < 0.001). Peaks in each type of genomic region were called via ASPeak. (B) Venn diagram showing overlap of DAZL iCLIP peaks in 3’ UTRs among 3 biological replicates. 1,670 replicated peaks (present in at least 2 of 3 replicates; highlighted in blue) were identified. After merging replicated peaks falling on consecutive nucleotides, 1,652 binding sites were identified. These binding sites correspond to 1,281 genes, which were designated as DAZL-bound genes. (C) GUU motif is enriched at DAZL 3’ UTR peaks. AME from the MEME Suite was used to identify motif enrichment at crosslinked nucleotides in replicated peaks relative to shuffled control sequences. (D-J) DAZL and IgG iCLIP gene tracks showing 3’ UTRs for *Taf1* (D), *Taf2* (E), *Taf4a* (F), *Taf4b* (G), *Taf7* (H), *Taf9b* (I), and *Tbp* (J). Each iCLIP track represents the crosslinked sites from the sum of unique reads from 3 biological replicates, with DAZL iCLIP reads in red and IgG iCLIP reads in black. The horizontal blue lines on top mark the replicated peaks.

DAZL iCLIP peaks in 3 biological replicates were identified in 3’ UTRs (**Fig 8A and 8B**), where DAZL binds to facilitate post-transcriptional regulation [12,13,31–34]. Defining DAZL binding sites as those iCLIP peaks in 3’ UTRs present in at least 2 of 3 biological replicates, we identified a total of 1,652 DAZL binding sites corresponding to 1,281 genes (**Fig 8B**). This is far fewer binding sites and targets than identified by Zagore et al. and Li et al. [12,13]. These differences may be due to the tissue used, as these studies used whole testes which contain multiple spermatogenic stages that express DAZL [12,13]. The 3’ UTR binding sites identified here were strongly enriched for the GUU motif with which DAZL preferentially interacts (**Fig 8C**) [12,13,35]. Replicated peaks were also identified in other genomic regions (S5**B Fig**, **S11 Table**), but the number of replicated peaks in these genomic regions was small and/or these peaks showed only moderate enrichment for DAZL’s GUU motif relative to the 3’ UTR peaks (**Fig 8B and 8C**, **S5B and S5C Fig**), suggesting that peaks outside of 3’ UTRs originate from nonspecific DAZL:RNA interactions.

Based on the 3’ UTR binding sites, DAZL targets 6 TFIID component genes, including *Taf4b* but not *Taf4a* (**Fig 8D–8J**, **S11 Table**). The observation that all of these genes exhibit reduced expression in *Dazl*-KO spermatogonia may reflect that DAZL protects these transcripts from degradation. Alternatively, the reduced TFIID gene expression in the *Dazl*-KO may reflect indirect regulation by DAZL, which may increase the protein expression of other transcription factors that are required for the expression of TFIID component genes.

**Fig 9:**
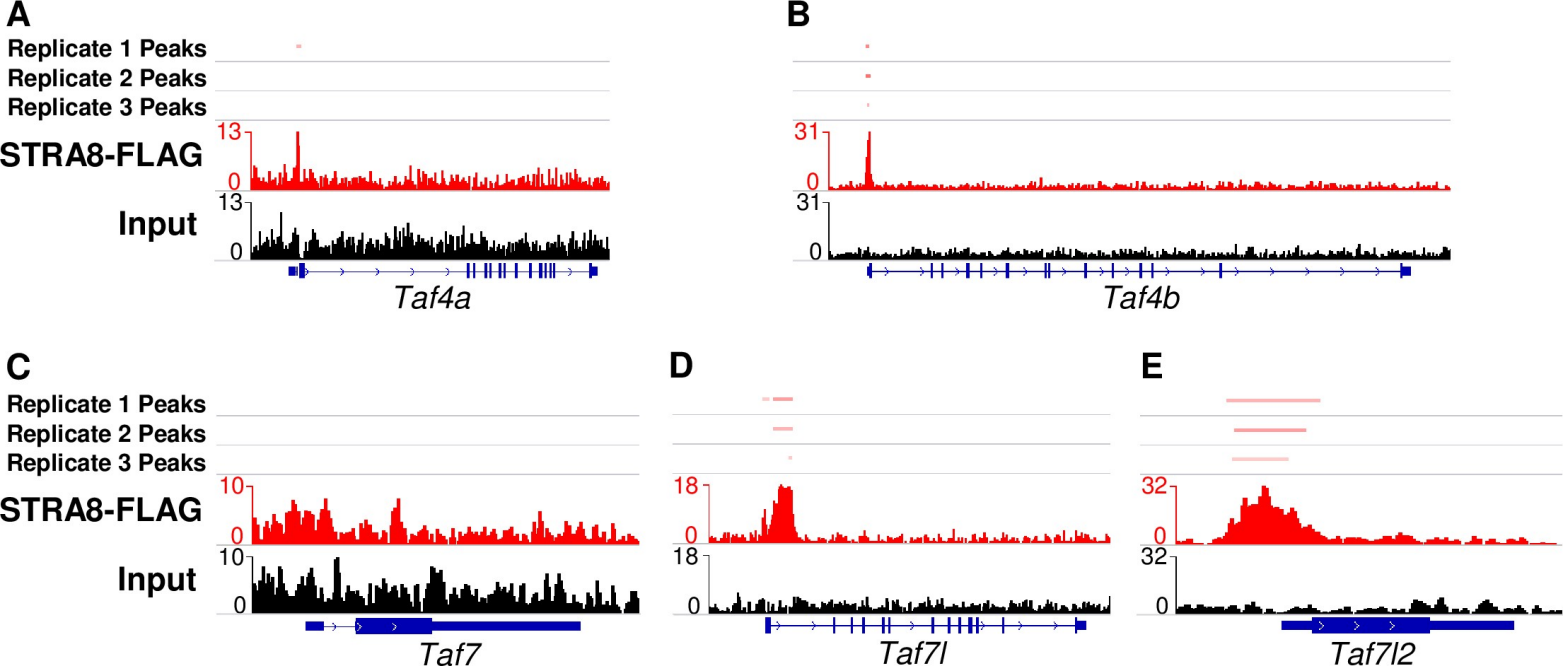
STRA8 ChIP-seq signal shows STRA8 binding at or near the promoters of TFIID subunits. (A-E) ChIP-seq signal from STRA8-FLAG samples and the peaks called (red) in comparison to input DNA (black) for *Taf4a* (A), *Taf4b* (B) *Taf7* (C), *Taf7l* (D), and *Taf7l2* (E).

To determine whether STRA8 directly promotes the gene expression of TFIID components, we reprocessed the ChIP-seq data of Kojima et al. [14] on preleptotene synchronized whole testis expressing a FLAG-tagged STRA8 protein. We found peaks in two of the three replicates at many of the same genes with reduced expression in *Stra8*-KO RNA-seq experiments, including *Taf4b*, *Taf7l*, and *Taf7l2*. By contrast, STRA8 does not bind to *Taf4a* or *Taf7*, which are not differentially expressed in the RNA-seq analysis (**Fig 9A–9E**, **S6A Fig**, **S12 Table**). Interestingly, STRA8-FLAG ChIP signal reliably localized to the 5’ UTR regions of *Taf4b* and *Taf7l2* (**Fig 9A** and **9E**), but localized after the first exon of *Taf7l* (**Fig 9D**). In addition to ChIP-seq data, in *in vitro* differentiated primordial germ cell-like cells (PGCLCs), treatment of PGCLCs with retinoic acid and bone morphogenetic protein 2 (BMP2) induces *Taf4b*, but not *Taf4a*, expression on culture day 9 (**S6B and S6C Fig**) [36]. However, this induction of *Taf4b* fails to occur if *Stra8* is knocked down in the cell culture system, which is also the case for *Taf7l* and *Taf7l2*, but not *Taf7* (**S6D–S6F Fig**). These combined lines of evidence including RNA-seq, ChIP-seq, and *in vitro Stra8* knockdown demonstrate a direct link between *Stra8* and several TFIID components in and around the time of meiotic initiation.

### SAGA complex subunit *Supt3* mirrors *Taf4b* regulation

**Fig 10:**
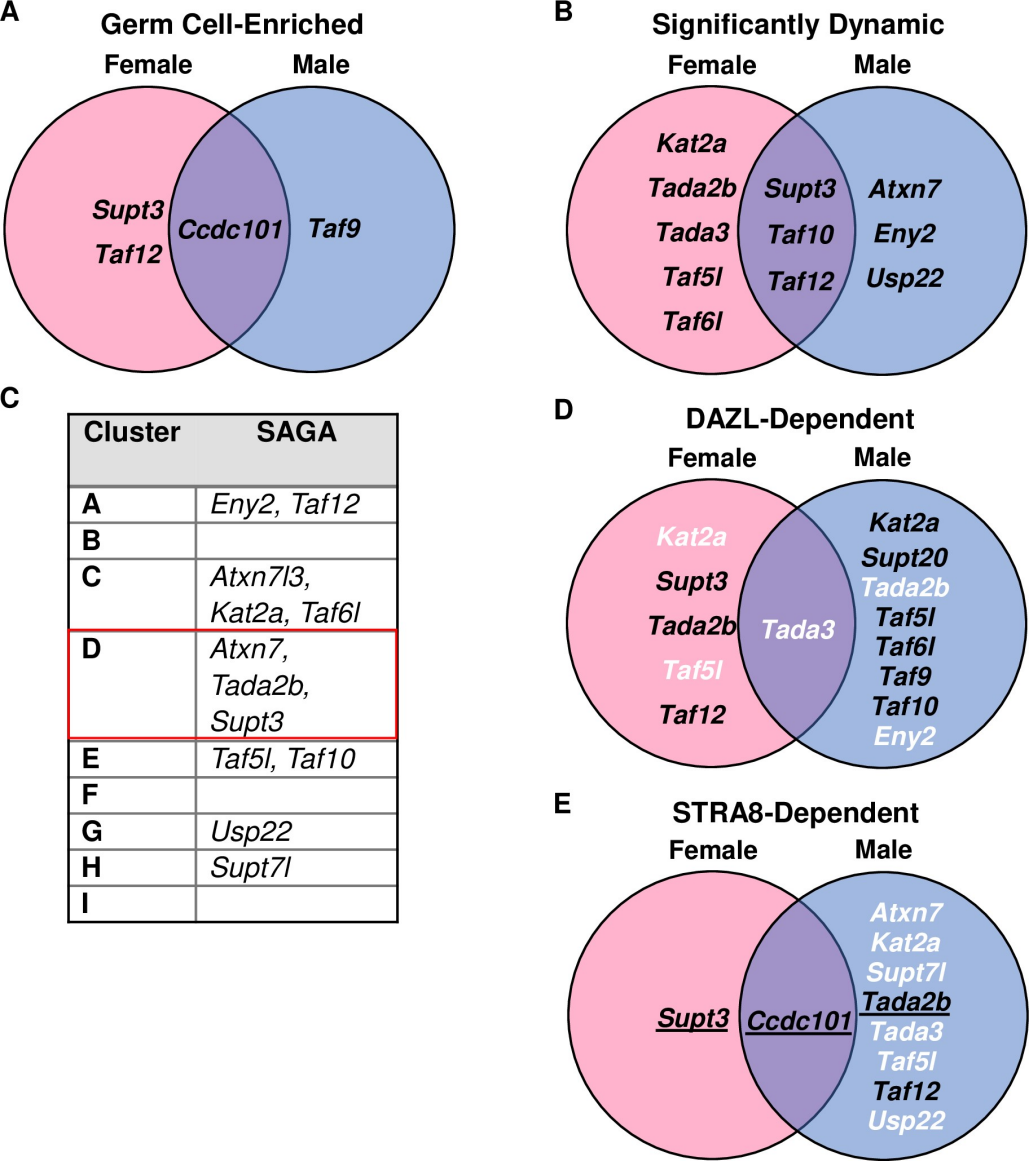
SAGA complex components not particularly germ cell relevant. (A) Venn diagram of SAGA subunits identified as significantly germ cell-enriched in the female and/or male germ cell time course data. (B) Venn diagram of SAGA subunits identified by ImpulseDE2 as significantly dynamic in the female and/or male germ cell time course data. (C) SAGA complex subunits that were part of the top 10,000 most variable genes in the k-means clustering. (D) Venn diagram of SAGA subunits found to be significantly (log_2_FC > |0.25|, p-adj < 0.05) different between the WT and *Dazl*-KO of the E14.5 ovary and/or PND6 spermatogonia. Black text indicates decreased mRNA expression in the *Dazl*-KO and white text indicates increased mRNA expression in the *Dazl*-KO compared to WT. Underlined text indicates the gene was found to have DAZL binding in its 3’ UTR in 2 out of 3 DAZL iCLIP replicates. (E) Venn diagram of SAGA found to be significantly (log_2_FC > |0.25|, p-adj < 0.05) different between the WT and *Stra8*-KO of the E14.5 ovary and/or male preleptotene germ cells. Black text indicates decreased mRNA expression in the *Stra8*-KO and white text indicates increased mRNA expression in the *Stra8*-KO compared to WT. Underlined text indicates peaks at transcription start sites detected in two of three ChIP-seq replicates.

As TFIID and Spt-Ada-Gcn5-acetyltransferase (SAGA) are closely related complexes, even sharing TAF9, TAF10, and TAF12 as subunits, we investigated whether these trends in germ cells were unique to TFIID or also applied to SAGA [10,37]. In the mouse time course data, we found four SAGA subunits (including *Taf12*) to be germ cell-enriched (**Fig 10A**) by the criteria of higher mRNA expression in the germ cells over the somatic cell samples for at least 3 of the 7 measured time points in either sex. ImpulseDE2 identified eleven SAGA subunits as significantly dynamic in female and/or male germ cells (**Fig 10B**) and *Atxn7*, *Tada2b*, and *Supt3* were found in Cluster D of our k-means clustering (**Fig 10C**, red box). When examining our data regarding *Dazl*, we found eleven subunits differentially expressed between WT and *Dazl*-KO samples in female and/or male mice, but only two of these genes (*Usp22* and *Trrap*) were identified as DAZL-bound in our iCLIP data and neither were found to be differentially expressed (**Fig 10D**, **S11 Table**). Similarly, we found ten SAGA genes to be differentially expressed between WT and *Stra8*-KO samples in female and/or male mice, but in the ChIP-seq data, only three of these genes display STRA8-FLAG peaks in their promoter regions (**Fig 10E**) and three more (*Taf5l*, *Taf9*, *Trrap*) were not differentially expressed. Altogether, only expression of *Supt3* is most similar to *Taf4b*, but its mRNA is not found to be DAZL-bound (**S11 Table**), which suggests that there are differences in how they are regulated during embryonic germ cell development.

## Discussion

The underlying transcriptional programs that drive germ cell development during mammalian embryogenesis are just coming into focus. In this study, we reprocessed publicly available RNA-seq datasets to understand the dynamics and potential regulators of the TFIID complex. We discovered that *Taf4b* is germ cell-enriched, significantly dynamic, clusters with meiotic genes, and is *Dazl*- and *Stra8*-regulated in female and male mice. Furthermore, our DAZL iCLIP data demonstrates that the 3’ UTRs of six TFIID components (*Taf1*, *Taf2*, *Taf4b*, *Taf7*, *Taf9b*, and *Tbp*) are directly bound by DAZL in leptotene spermatocytes. Moreover, reprocessing of STRA8-FLAG ChIP-seq data indicates that six TAFs (*Taf3*, *Taf4b*, *Taf7l*, *Taf7l2*, *Taf9*, and *Taf11*) have STRA8 bound to their promoters in preleptotene spermatocytes [13,14]. Of all these genes that were either DAZL- or STRA8-regulated, only *Taf4b*, *Taf7l*, and *Taf9b* were also germ cell-enriched, significantly dynamic in their expression, and clustered with meiotic genes. Taken together, these data indicate that several TAFs may participate in germ cell development, particularly during early meiosis, and potentially implicate a germ cell-specific TFIID complex that is preferentially stabilized to integrate proper germ cell-specific transcription and development.

Although basal transcription components such as TFIID are not widely studied in the context of reproduction, Sisakhtnezhad and Heshmati [38] found that *Taf7l* (with *Dazl*, *Mael*, etc.) was one of the 30 most significantly SSC-enriched genes in comparison to mesenchymal stem cells in PND7 mice. In addition, *Taf1*, *Taf2*, *Taf4b*, *Taf7*, *Taf7l2*, and *Taf9b* were also significantly enriched in SSCs along with other basal transcription machinery such as RNAPII subunits [38]. Soh et al. found that *Taf4b*, *Taf5*, *Taf7l*, *Taf7l2*, and *Taf9b* were “ovarian germ cell enriched”, of which *Taf7l*, *Taf7l2*, and *Taf9b* are also highlighted as “meiotic prophase” genes [25]. It is already known that *Taf7l* is essential for male fertility: *Taf7l*-KO male mice are infertile due to defects in spermiogenesis [18]. *Taf7l2* was only recently uncovered as a rodent-specific *Taf7l* retrogene [14] and it is not known if there is a fertility defect in a *Taf7l2* mutant. *Taf7l* interacts with *Trf2* (also known as *Tbpl1*) to bind to promoters of genes in mouse testis. Interestingly, *Trf2/Tbpl1* was found to be significantly dynamic in female germ cells, but not germ cell-enriched nor significantly affected by *Dazl* or *Stra8* in our data. It might be that *Taf7l* interacts with different proteins to perform a different role in the embryonic ovary or postnatal testis. E13.5 germ cells have been found to have much higher transcription levels than their somatic cell counterparts, and this has been proposed to be a phase of hypertranscription in the germ cells [39]. However, this phase was found to end at E15.5, which contrasts with many of the TAFs we observed that increase in gene expression at E15.5.

*Taf9b* participating in the Cluster D in the k-means clustering was surprising out of the five TAFs found in that cluster. *Taf9b* is known to play a critical role in proper differentiation of motor neurons, but it has no known role in germline development, as *Taf9b*-KO mice are viable and fertile [40]. *Taf9b* is not the only gene to be associated with cell type-specific differentiation. *Taf7l* has also been found to play a role in adipocyte formation [41,42]. When the germ cell samples were split into female- or male-only for individual analysis, *Taf4b*, *Taf7l*, *Taf7l2*, and *Taf9b* remained clustered together and with other meiotic genes in female germ cells. However, in male germ cells, these four TAFs did not cluster together, but *Taf4b* and *Taf7l2* did, as well as *Taf7l* and *Taf9b* (**S7 Table**). Hill et al. also grouped *Taf7l*, *Taf7l2*, and *Taf9b* together as germline reprogramming responsive (GRR) genes, which is a group of 45 genes that includes *Dazl*, *Hormad1*, and thirteen other genes that were also found in Cluster D [43]. These genes were designated GRR because their promoters were highly methylated at E10.5 but this DNA methylation was reduced during germline epigenetic programming, and the genes became progressively more transcribed in both sexes. This consistent association with essential gamete generation and meiotic genes in both our clustering analysis and other published research strongly implicates *Taf4b*, *Taf7l*, *Taf7l2*, and *Taf9b* as genes that warrant further study. Only TAF4b and TAF7l are being studied for their germ cell-specific function. It is unknown to what extent, if any, these other TAFs regulate the expression of each other. Future research should investigate if genes such as *Taf7l* are differentially expressed in the *Taf4b*-deficient mouse. Our finding that, among the SAGA complex genes, only *Supt3* (also known as *Spt3*) is germ-cell enriched, significantly dynamic, in Cluster D, and STRA8-regulated suggests that this gene may function in germline-specific gene expression. Despite not being DAZL-regulated, it is possible that the SAGA complex could play a role in germ cell development in a similar way to *Taf4b*.

Our findings also reinforce the role of *Taf4b* in germ cell development, but now as part of a larger, coordinated effort with additional TFIID components. Given that previous research has shown that TAF4b functions during meiosis and localizes to the promoters of *Dazl* and *Stra8* at E18.5, it is possible that TAF4b, DAZL, STRA8, and perhaps other factors work interdependently. Identifying DAZL and STRA8 as regulators of *Taf4b* is an important step forward in understanding *Taf4b*, but nevertheless, there are likely more regulators yet to be found. This is hinted at in our data, where male *Taf4b* expression rises at E15.5 (**Fig 2A and 2B**) in the absence of STRA8. It is plausible that another transcription factor facilitates this increase. As STRA8 is considered a transcriptional amplifier rather than an activator, it is likely that this unknown transcription factor(s) is shared between female and male germ cells. Future work in embryonic germ cells, particularly in males, should add more context to the role TAF4b may be playing there.

The specialized role for TAF4b as a germ cell relevant TFIID component is bolstered by single-cell RNA-seq analyses in mice and humans that associate *Taf4b* with essential developmental steps in *Id4*-GFP adult mouse germ cells and adult human spermatogonia [44]. Alternative Taf4 subunits have been identified in both *Drosophila melanogaster* (known as *nht*) and *Arabidopsis thaliana* (known as taf4b) [45,46]. The alternative Taf4 genes in vertebrates, *Drosophila*, and *Arabidopsis* arose independently [10]. Interestingly, *Drosophila nht* is testis-specific and required for male fertility, and Arabidopsis Taf4b is directly implicated in regulating meiotic crossover rates and germline transcription, similar to mouse *Taf4b* [47]. This mechanism of an alternative Taf4 in TFIID, particularly for the purpose of germ cell development and function, is an intriguing instance of convergent evolution across plants, invertebrates, and vertebrates.

TFIID has been typically viewed as static and generic in composition as a basal transcription factor. These dynamic gene expression data, particularly for *Taf4b*, challenge this view and suggest that there are subunits in TFIID that are preferred and regulated over others in mammalian germ cells. This may also be the case for other cell type-specific differentiation programs, as some of the same variant TFIID subunits shown to be expressed in a germ cell-specific manner here have also been shown to regulate somatic cells, including neurons and adipocytes. Though initially unexpected, such research indicates that there is more information to be garnered from studying TFIID in the context of germ cells and other specialized cell types.

## Materials & methods

### Ethics statement

This study was approved by Brown IACUC protocol #1803000344. For the iCLIP experiment, the MIT IACUC approved this research (#067-059-20). The primary method of euthanasia is CO_2_ inhalation and the secondary method used is cervical dislocation both as per AVMA guidelines on euthanasia.

### Mice

Mice that were homozygous for an *Oct4-EGFP* transgene (The Jackson Laboratory: B6;129S4-*Pou5f1*^*tm2Jae*^/J) were mated for mRNA and protein collections. Timed matings were estimated to begin at day 0.5 by evidence of a copulatory plug. To confirm the sex of E12.5 and younger embryos, genotyping for the presence or absence of the *Sry* gene was completed. Male and female embryos were identified at E13.5 and older by confirming the presence or absence of testicular cords. Genomic DNA from tails was isolated using ThermoFisher PureLink Genomic DNA isolation kit (Cat #: K182001) for PCR genotyping assays. All animal protocols were reviewed and approved by Brown University Institutional Animal Care and Use Committee and were performed in accordance with the National Institutes of Health Guide for the Care and Use of Laboratory Animals. Gonads were dissected out of embryos into cold PBS. Wildtype C57BL/6N mice (Taconic Biosciences) were mated to produce postnatal males for iCLIP collection. All experiments involving mice were performed in accordance with the guidelines of the Massachusetts Institute of Technology (MIT) Division of Comparative Medicine, which is overseen by MIT’s Institutional Animal Care and Use Committee (IACUC). The animal care program at MIT/Whitehead Institute is accredited by the Association for Assessment and Accreditation of Laboratory Animal Care, International (AAALAC), and meets or exceeds the standards of AAALAC as detailed in the Guide for the Care and Use of Laboratory Animals. The MIT IACUC approved this research (no. 0617-059-20).

### Embryonic gonad dissociation and fluorescence-activated cell sorting

To dissociate gonadal tissue into a single-cell suspension, embryonic gonads at specified times were harvested and placed in 0.25% Trypsin/EDTA and incubated at 37°C for 15–20 minutes. Eppendorf tubes were flicked to dissociate tissue halfway through and again at the end of the incubation. Trypsin was neutralized with 75 μL of FBS. Cells were pelleted, the supernatant was removed, and cells were resuspended in 100 μL PBS. The cell suspension was strained through a 35 μm mesh cap into a FACS tube (Gibco REF# 352235). Propidium iodide (1:500) was added to the cell suspension as a live/dead distinguishing stain. Fluorescence-activated cell sorting (FACS) was performed using a Becton Dickinson FACSAria III in the Flow Cytometry and Cell Sorting Core Facility at Brown. A negative control of a non-transgenic mouse gonad was used for each experiment to establish an appropriate GFP signal baseline. Dead cells were discarded based on propidium iodide signal and then cells were sorted at 4°C in PBS based on GFP signal into GFP^+^ or GFP^-^ samples. Cells for western blots were pelleted, PBS was removed, flash-frozen with liquid nitrogen, and stored at -80°C.

### Western blotting

Protein to be loaded for western blots was estimated based on cell numbers reported during FACS. Thawed cells were mixed with laemmli sample buffer and β-mercaptoethanol, pipetted thoroughly, incubated on ice for 1 hour, and stored at -80°C. Western blotting was performed by loading roughly similar amounts of protein (estimated based on cell number) into a BioRad Mini-PROTEAN TGX 4–15% gel (Cat. #456–1083). Protein was transferred to nitrocellulose membrane (0.45 μm). Nitrocellulose was cut, blocked in 5% milk in PBS with 0.1% Tween (PBS-T) or blocked in 5% Amersham ECL Prime Blocking Reagent (RPN418) for 30 minutes at room temperature. Incubation in primary antibody in 1% milk in PBS-T or only PBS-T was performed in 4°C overnight and secondary antibody in 1% milk in PBS-T or only PBS-T for 1 hour at room temperature. In between the block/primary and primary/secondary incubations, nitrocellulose was washed in PBS-T at least three times for 5 minutes. β-Actin or GAPDH was used as a loading control. GE Healthcare Amersham ECL Prime Western Blotting Detection Reagent (RPN2232) was used for horseradish peroxidase-tagged (HRP) secondary antibody detection. For stripping antibodies, Restore Western Blot Stripping Buffer (ThermoFisher: 21059) was used for 10 minutes at room temperature. For relative protein quantification, ImageJ was used to normalize protein signal to β-Actin in each cell type.

Antibodies used were as follows: mouse monoclonal anti-TAF_II_135 (BD Transduction Laboratories: 612054), polyclonal rabbit anti-mouse TAF4B (as described previously [11]), mouse monoclonal TAF7 (Novus Biologicals: H00006879-M01), rabbit polyclonal Taf7l (a generous gift from Dr. Haiying Zhou and Dr. Robert Tjian, rabbit polyclonal MVH (Abcam: ab13840); mouse monoclonal β-Actin (Ambion: AM4302).

### RNA-seq data analysis

All computational scripts regarding RNA-seq used in this publication are available to the public: https://github.com/mg859337/TAF_Manuscript_MG/tree/master/RNAseq. Datasets SRP059601, SRP059599, SRP057098, SRP045294, SRP049981, SRP128645, and SRP150721 were from NCBI SRA, and dataset E-MTAB-4616 was obtained from ArrayExpress. Kojima et al. performed RNA-seq on four Stra8-WT samples, two labeled “high-Stra8” and two “low-Stra8”, but only the “high-Stra8” were used as WT in these data because the “low-Stra8” were in early preleptotene and not representative of full meiotic initiation in male germ cells. All accessed raw fastq files were initially processed on Brown University’s high-performance computing cluster at the Center for Computation and Visualization. They were analyzed using FastQC (v0.11.4 or 0.11.5) for quality and then aligned to the mm10 or hg38 genomes using HiSat2 (v2.1.0) with the optional -dta setting enabled (**S12 Table**) [48,49]. Resulting sam files were converted to bam files using Samtools (v1.9) [50].

To obtain transcripts per million (TPMs) for each sample, StringTie (v1.3.3b) was used with the optional parameters -A and -e [49]. A gtf file for each sample was downloaded and, using RStudio (R v3.5.1), TPMs of all samples were aggregated into one comma separated (csv) file using a custom R script. To create interactive Microsoft Excel files for exploring the TPMs of each dataset: the csv of aggregated TPMs was saved as an Excel spreadsheet, colored tabs were added to set up different comparisons, and a flexible Excel function was created to adjust to new gene name inputs. To explore the Excel files, please find the appropriate tab and type in the gene name of interest into the highlighted yellow boxes. There is an Excel file for each dataset analyzed in the supplementary tables.

To obtain count tables, HTSeq (v0.9.1) and the count tables for each dataset were merged using a custom RStudio script [51]. Metadata files for dataset were created manually in Excel and saved as a csv. These count tables were used to create PCA plots by variance-stabilizing transformation (vst) of the data in DESeq2 (v1.22.2) and plotting by ggplot2 (v3.1.0) [52,53]. DESeq2 was also used for differential gene expression analysis, where count tables and metadata files were used as input.

ImpulseDE2 (v1.6.1), a Bioconductor R package that can identify differentially expressed genes in longitudinal count datasets, was used to identify “significantly dynamic” genes from E9.5 –E18.5 female and male Oct4-EGFP^+^ germ versus Oct4-EGFP^-^ somatic cell time course datasets [24]. ImpulseDE2 was used in case-only mode and no batch effects settings were added to the parameters.

For k-means clustering, iDEP (v.9) was used. It is a webpage hosted by South Dakota State University that is a simple and fast resource to perform typical RNA-seq data analysis [54]. The Sangrithi et al. (2018) mouse time course merged counts table was uploaded to iDEP. Counts were filtered out by the criteria of at least 0.5 counts per million in one of the samples. The data was then transformed using vst, in the “Heatmap” module the blue-white-red color scheme was chosen and in the “k-Means” clustering module the top 10,000 most variable genes across the time course were selected for clustering. The setting of 9 clusters was chosen using the within-sum-of-squares (wss) elbow method displayed under the “How many clusters?” button. The heatmap of gene clustering and associated list of clustered genes were downloaded. Gene ontology is another part of the “k-Means” module, the GO category of Biological Process was chosen, and the enrichment details were downloaded.

### iCLIP library construction, sequencing, and computational analysis

To obtain testes enriched for the leptotene stage of meiotic prophase I, spermatogenesis was chemically synchronized via a protocol originally developed by Hogarth et al. and modified by Romer et al. [55,56]. Briefly, on PND2-8, male mice were injected daily subcutaneously with WIN 18,446 (Santa Cruz Biotechnology) at 0.1 mg/gram body weight. On PND9, the mice were injected once subcutaneously with retinoic acid (RA; MilliporeSigma) at 0.0125 mg/gram body weight. Mice were euthanized 8.0 days after the RA injection to obtain testes enriched for leptotene spermatocytes. From each pup, one half of a testis was collected as a biopsy for histological analysis to verify enrichment of leptotene spermatocytes, and the remaining 1.5 testes were collected for iCLIP. For histological analysis, the testis biopsy was fixed in Bouin’s solution for 3h at room temperature. Testis sections were stained with anti-STRA8 (rabbit polyclonal; Abcam ab49405) and then counterstained with hematoxylin, as previously described [14]. At least 40 seminiferous tubule cross-sections per mouse were analyzed for morphology [30] and STRA8 expression, found in preleptotenes and early leptotenes [57,58]. For all samples analyzed via iCLIP, 100% of the tubule cross-sections contained leptotene spermatocytes but not preleptotene or zygotene spermatocytes.

After synchronization of spermatogenesis, testis tubules were dissociated by pipetting in ice-cold PBS and irradiated three times at 200 mJ/cm^2^ at 254 nm in a Stratalinker 2400 before pelleted, flash frozen, and stored at -80°C until library preparation. DAZL and IgG iCLIP libraries were prepared as previously described [27]. For each biological replicate, 1.5 testes from a single mouse were lysed in 640 μl lysis buffer. Immunoprecipitations were carried out via anti-DAZL (rabbit polyclonal; Abcam ab34139; validated for CLIP in studies [12,13]) or polyclonal rabbit IgG (Abcam ab27478) using 12 μg antibody bound to 120 μl Dynabeads Protein G (ThermoFisher Scientific 10003D) with 600 μl lysate. iCLIP libraries were prepared in two batches, with each batch containing an equal number of DAZL iCLIP and IgG iCLIP libraries. A total of 3 DAZL iCLIP and 3 IgG iCLIP libraries were generated. The libraries were pooled and sequenced with 50bp single-end reads on the Illumina HiSeq 2500 machine. The 5’ end of each iCLIP read contained (in 5’ to 3’ orientation), a 3-nt random barcode, a 4-nt sample-specific barcode, and a 2-nt random barcode.

All computational scripts used in this publication for the iCLIP analysis are available at: https://github.com/mmikedis/Gura_et_al_2019_PLOS_Genetics. Reads were quality-trimmed with cutadapt v1.8 (options: -q 20 -m 24) [59]. PCR duplicates were collapsed using the FASTX-Toolkit v0.0.14 (http://hannonlab.cshl.edu/fastx_toolkit/index.html), and the 5'-most 3-nt random barcode was trimmed from the 5' end of each read with the fastx_trimmer tool from FASTX-Toolkit v0.0.14 (options: -f 4). Reads were demultiplexed using the fastx_barcode_splitter.pl tool from FASTX-Toolkit v0.0.14 (options:—bol), and the sample-specific barcodes with remaining random barcodes were trimmed from the 5’ end of each read with the fastx_trimmer tool (options: -f 7). iCLIP libraries were mapped to the mouse genome (mm10 assembly) via STAR v2.5.4b [60] (options:—outFilterMultimapNmax 1—alignEndsType Extend5pOfRead1—outFilterMismatchNmax 2—outSAMattributes None—outReadsUnmapped Fastx). All other parameters were set to default. The iCLIP mapped reads were then converted to crosslinked nucleotides (i.e., the nucleotide immediately preceding the first nucleotide of the mapped read) using a custom script [61]. Within each library batch, DAZL crosslinked peaks were called using the DAZL iCLIP crosslinked nucleotides via ASPeak v2.0.0 [62] with the IgG iCLIP crosslinked nucleotides for the -control parameter. All other parameters were set to default. The UCSC RefSeq transcript annotations and Retrogenes V6 annotations for the mm10 assembly were used to call peaks. Given that DAZL binds the 3’ UTR of coding transcripts, peaks were called via the following hierarchy: 3' UTR exon > 5' UTR exon > coding exon > ncRNA > retrogene > intron > intergenic region. Peaks were filtered for p < 0.001, and then replicated peaks present in at least 2 out of 3 biological replicates were identified. Replicated peaks identified in consecutive nucleotides were merged into a single peak. iCLIP sequencing data and analysis have been deposited in NCBI GEO under accession number GSE139005 and NCBI SRA under accession number SRP226023.

Enrichment of the GUU motif at replicated peaks from each genomic region was assessed via the MEME Suite’s AME v4.11.2 [63] (options:—scoring avg—method ranksum) using the replicated crosslinked nucleotides ±2 nt and shuffled control sequences.

### ChIP-seq data analysis

All computational scripts used in this publication regarding ChIP-seq are available to the public: https://github.com/mg859337/TAF_Manuscript_MG/tree/master/ChIPseq. ChIP-seq samples from dataset SRP150721 was obtained from NCBI SRA and all raw fastq files from genotype FLAG/FLAG were initially processed on Brown University’s high-performance computing cluster at the Center for Computation and Visualization. They were analyzed using FastQC (v0.11.5) for quality and trimmed for very low-quality reads using Trim Galore! (v0.5.0) (https://www.bioinformatics.babraham.ac.uk/projects/trim_galore/).

These trimmed reads were then aligned to the mm10 genome using Bowtie2 (v 2.3.0) (**S12 Table**) [64]. Resulting sam files were converted to bam files, then unmapped and duplicated reads were removed using Samtools (v1.9) [50]. Bedtools (v2.26.0) was used to removed regions in bam files that mapped to ChIP-seq blacklisted regions in the mm10 genome [65]. For visualization, Samtools was used to merge replicates and Integrative Genomics Viewer (IGV) was used to visualize the gene tracks [66]. For calling peaks MACS (v 2.1.1) was used, comparing the FLAG replicate to its IgG control [67].

## Supporting information

S1 Fig Replicates of western blots and protein signal of TAF4b in cells sorted from female E13.5 Oct4-EGFP gonads. (A-B) Entire blots of images displayed in Fig 2E of TAF4a (135 kDa), TAF4b (105 kDa), and β-Actin (42 kDa) (A) and MVH (76 kDa) after antibody stripping and reprobing (B). (C) Entire blot of second western blot replicate of E13.5 Oct4-EGFP sorted gonads. Four female mice and three male mice were used to load roughly 40,000 cells. (D) Entire blot of third western blot replicate of E13.5 Oct4-EGFP sorted gonads. Four female mice and three male mice were used in the experiment to load roughly 25,000 cells. (E) Approximate numbers of female GFP^-^ and GFP^+^ cells loaded into western blot are as indicated. TAF4b protein signal is detected in only the GFP^+^ lane (germ cell), despite 5X more cells being loaded into the GFP^-^ lane. β-Tubulin is a protein loading control. S1 Fig is associated with Fig 2.
(PDF)

S2 Fig Independent RNA-seq datasets replicate increase in *Taf4b* mRNA expression after E13.5 in both female and male mouse germ cells and low expression of *Taf4a*. (A-B) RNA-seq from Miyauchi et al. [Supporting Information References 1] of cells sorted from Stella-EGFP E9.5-E13.5 and MVH-RFP E14.5-E15.5 female (A) and male (B) mice. For E9.5-E11.5, sexes were pooled together and the same data appear in both plots. (C-D) RNA-seq from Seisenberger et al. [Supporting Information References 2] of cells sorted from Oct4-EGFP female (C) and male (D) mice. For E11.5, sexes were pooled together, and the same data point appears in both plots. S2 Fig is associated with Fig 2.
(PDF)

S3 Fig Germ cell-enriched TFIID subunits and closer examination of *Taf7*, *Taf7l*, and *Taf7l2*. (A) Removal of *Taf7l* expression to more closely examine female germ cell expression of *Taf7* and *Taf7l2* over time. (B) Removal of *Taf7* expression to more closely examine male germ cell expression of *Taf7l* and *Taf7l2* over time. Expression of female (C, E, G) and male (D, F, H) mRNAs of *Taf7*, *Taf7l*, and *Taf7l2* in germ cells (“G”) and somatic cells (“S”) from E9.5 to E18.5 (* = log_2_FC > |0.25|, p-adj. < 0.05). (I-J) *TAF7* and *TAF7L* expression in human gonads indicate that *TAF7L* is barely detectable at 7 weeks post-fertilization. *Taf7l2* does not have a human homolog. (K-L) From ~8 to ~16 weeks post-fertilization in females and to ~20 weeks post-fertilization in males, human *TAF7L* mRNA expression is low and unchanging while *TAF7* expression is variable over time. This is dissimilar to the mouse *TAF7L* RNA-seq data. Error bars indicate ± SEM. (M) Venn diagram of TFIID subunits identified as significantly germ cell-enriched in the female and/or male germ cell time course data. S3 Fig is associated with Fig 2, Fig 3 and Fig 4.
(PDF)

S4 Fig Within sum of squares (wss) graph for evaluating how many clusters to set in k-means clustering. Nine clusters were chosen. S4 Fig is associated with Fig 5 and Table 1.
(PDF)

S5 Fig DAZL iCLIP in testes synchronized for leptotene spermatocytes. (A) Radioblot of DAZL:RNA complexes from postnatal testes synchronized for leptotene spermatocytes. DAZL:RNA complexes are larger than 37 kDa, the approximate molecular weight of DAZL. One of three biological replicates used to prepare iCLIP libraries reported here. (B) Venn diagram showing overlap of DAZL iCLIP peaks among 3 biological replicates in genomic regions other than the 3’ UTR. (C) Enrichment of DAZL’s GUU motif at replicated iCLIP peaks from genomic regions other than the 3’ UTR. AME from the MEME Suite was used to identify motif enrichment at crosslinked nucleotides in replicated peaks relative to shuffled control sequences. S5 Fig is associated with Fig 8.
(TIF)

S6 Fig STRA8 peaks in preleptotene germ cells and knockdown experiments in primordial germ cell-like cells (PGCLCs). (A) STRA8-FLAG peaks called by MACS2 at the transcription start sites of TFIID components in comparison to the DNA input control. (B-F) mRNA expression levels in WT and *Stra8*-knockdown in vitro differentiated PGCLCs from reprocessed Miyauchi et al. RNA-seq using retinoic acid and BMP2. Expression of *Taf4a* (B) and *Taf7* (D) do not see a strong induction at culture day 9 (c9) nor are differentially expressed in the *Stra8*-knockdown. *Taf4b* (C), *Taf7l* (E), and *Taf7l2* (F) are highly expressed at c9 but fail to be induced in *Stra8*-knockdown cells (* = log_2_FC > |0.25|, p-adj. < 0.05). S6 Fig is associated with Fig 7.
(PDF)

S1 Table DESeq2 output for comparisons between germ and somatic cells at different time points. Sheets contain DESeq2 results for germ versus somatic cell gene expression for every time point and sample type. S1 Table is associated with Fig 1, Fig 2, Fig 4, and S3 Fig.
(XLSX)

S2 Table Transcripts per million (TPM) of all reprocessed time course data from Sangrithi et al. with tabs for asking specific questions about the data. Sheet 2 (“All_TPMs”) contains a TPM values table for all samples and genes. Sheets 3–10 contain search and plotting tools to quickly evaluate the data. S2 Table is associated with Fig 1, Fig 2, Fig 4, and S3 Fig.
(XLSX)

S3 Table Transcripts per million (TPM) of all reprocessed time course data from Gkountela et al. with tabs for asking specific questions about the data. Sheet 2 (“All_TPMs”) contains a TPM values table for all samples and genes. Sheets 3–5 contain search and plotting tools to quickly evaluate the data. S3 Table is associated with Fig 3 and S3 Fig.
(XLSX)

S4 Table Transcripts per million (TPM) of all reprocessed time course data from Tang et al. with tabs for asking specific questions about the data. Sheet 2 (“All_TPMs”) contains a TPM values table for all samples and genes. Sheet 3 (“Quick_Calc”) contains search and plotting tools to quickly evaluate the data. Sheet 4 (‘DESeq_PGCs_Somatic’) contains DESeq2 results for human female PGC versus gonadal somatic gene expression. S4 Table is associated with Fig 3 and S3 Fig.
(XLSX)

S5 Table Transcripts per million (TPM) of all reprocessed time course data from Irie et al. with tabs for asking specific questions about the data. Sheet 2 (“All_TPMs”) contains a TPM values table for all samples and genes. Sheet 3 (“Quick_Calc”) contains search and plotting tools to quickly evaluate the data. Sheet 4 (‘DESeq_PGC_Somatic’) contains DESeq2 results for human male PGC versus gonadal somatic gene expression. S5 Table is associated with Fig 3 and S3 Fig.
(XLSX)

S6 Table ImpulseDE2 output for individual sex and cell type data. Sheets 2–5 correspond to the ImpulseDE2 output for female germ cells, male germ cells, female somatic cells, and male somatic cells, respectively. S6 Table is associated with Fig 4.
(XLSX)

S7 Table k-means clustering and gene ontology of clusters. Sheet 2 (‘Kmeans_10000’) contains k-means clustering output for the 10,000 most variable genes across all germ cells samples in the Sangrithi et al. reprocessed time course data, 9 clusters total. Sheet 3 (‘GO_Biological_Process) contains the significant gene ontology enrichment for all clusters. Sheets 4 and 6 (‘Kmeans_10000_Females_Only’ and ‘Kmeans_10000_Males_Only’) are the k-means clustering output for the 10,000 most variable genes in germ cell samples, 9 clusters total from female and male samples, respectively. Sheets 5 and 7 (‘GO_Biological_Process_Females’ and ‘GO_Biological_Process_Males’) are the gene ontology for the female- and male-only k- means clustering, respectively. S7 Table is associated with Fig 5, Table 1, and S5 Fig.
(XLSX)

S8 Table Transcripts per million (TPM) of all reprocessed RNA-seq data from Soh et al. E14.5 females with tabs for asking specific questions about the data. Sheet 2 (“All_TPMs”) contains a TPM values table for all samples and genes. Sheet 3 (“Quick_Calc”) contains search and plotting tools to quickly evaluate the data. Sheets 4 and 5 (‘DESeq_WT_DazlKO’ and ‘DESeq_WT_Stra8KO’) contain DESeq2 results for WT versus KO gene expression. S8 Table is associated with Fig 6, Fig 7, and Fig 8.
(XLSX)

S9 Table Transcripts per million (TPM) of all reprocessed RNA-seq data from Zagore et al. PND6 spermatogonia with tabs for asking specific questions about the data. Sheet 2 (“All_TPMs”) contains a TPM values table for all samples and genes. Sheet 3 (“Quick_Calc”) contains search and plotting tools to quickly evaluate the data. Sheet 4 (‘DESeq_WT_DazlKO’) contains DESeq2 results for WT versus KO gene expression. S9 Table is associated with Fig 6 and Fig 8.
(XLSX)

S10 Table Transcripts per million (TPM) of all reprocessed RNA-seq data from Kojima et al. male preleptotene germ cells with tabs for asking specific questions about the data. Sheet 2 (“All_TPMs”) contains a TPM values table for all samples and genes. Sheet 3 (“Quick_Calc”) contains search and plotting tools to quickly evaluate the data. Sheet 4 (‘DESeq_WT_Stra8KO’) contains DESeq2 results for WT versus KO gene expression. S10 Table is associated with Fig 7 and Fig 8.
(XLSX)

S11 Table Information regarding iCLIP libraries and replicated peaks identified in DAZL iCLIP. Sheet 2 contains read numbers from the iCLIP libraries. Sheets 3–9 contain the replicated iCLIP peaks identified in the following genomic regions: 3’ UTR (Sheet 3), 5’ UTR (Sheet 4), CDS (Sheet 5), ncRNA (Sheet 6), retrogene (Sheet 7), intron (Sheet 8), and intergenic (Sheet 9). S11 Table is associated with Fig 8 and S5 Fig.
(XLSX)

S12 Table Narrowpeaks called from all reprocessed ChIP-seq data from Kojima et al. male preleptotene-synchronized testis germ cells. Each sheet (2–7) represents the MACS narrowpeak file output for each sample. S12 Table is associated with Fig 7, Fig 8, and S6 Fig.
(XLSX)

S13 Table Information regarding the sequencing samples reprocessed in this manuscript. Sheet 2 (‘Alignment_Details_RNAseq’) includes sample names, reads, single vs paired end, and alignment rate for all reprocessed RNA-seq samples. Sheet 3 (‘Alignment_Details_CHIPseq’) includes sample names, reads, single vs paired end, and alignment rate for all reprocessed ChIP-seq samples. S13 Table is associated with the Materials & Methods.
(XLSX)
