# Supplementary figures and images for: Dynamic and regulated TAF gene expression during mouse embryonic germ cell development
Source: PLoS Genet. 2020 Jan 8;16(1):e1008515. doi: 10.1371/journal.pgen.1008515 (PMC7010400; doi:10.1371/journal.pgen.1008515)

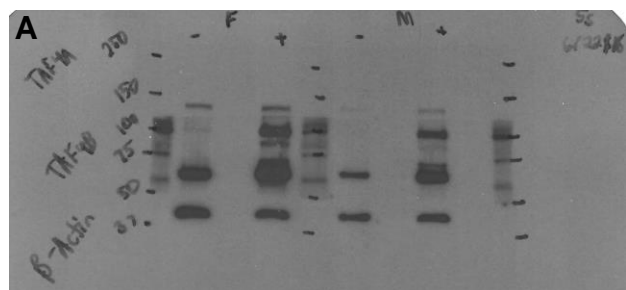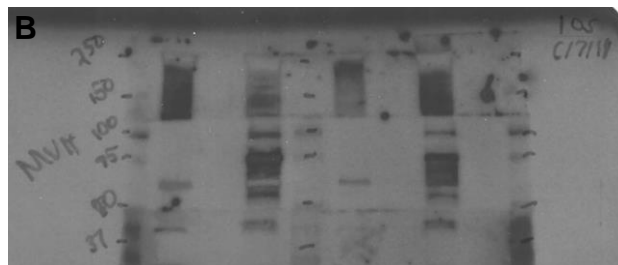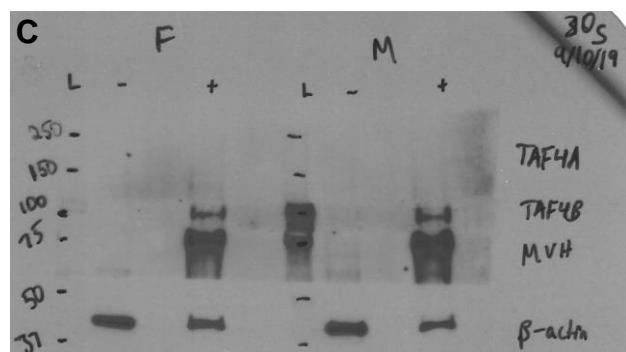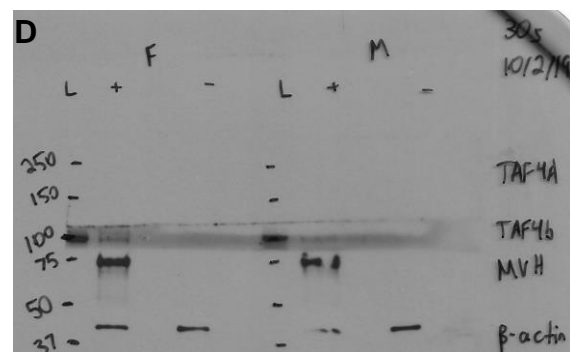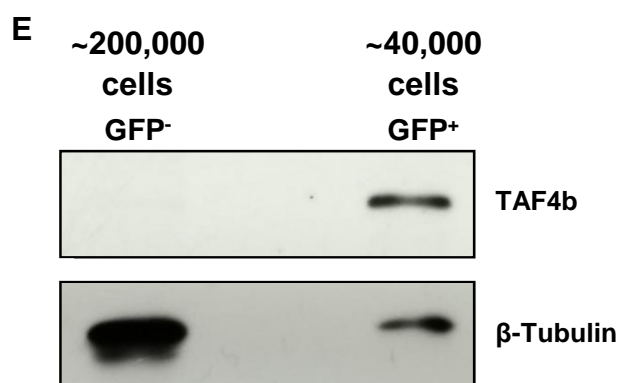

Supplement: S1 Fig — (A-B) Entire blots of images displayed in Fig 2E of TAF4a (135 kDa), TAF4b (105 kDa), and β-Actin (42 kDa) (A) and MVH (76 kDa) after antibody stripping and reprobing (B). (C) Entire blot of second western blot replicate of E13.5 Oct4-EGFP sorted gonads. Four female mice and three male mice were used to load roughly 40,000 cells. (D) Entire blot of third western blot replicate of E13.5 Oct4-EGFP sorted gonads. Four female mice and three male mice were used in the experiment to load roughly 25,000 cells. (E) Approximate numbers of female GFP- and GFP+ cells loaded into western blot are as indicated. TAF4b protein signal is detected in only the GFP+ lane (germ cell), despite 5X more cells being loaded into the GFP- lane. β-Tubulin is a protein loading control. S1 Fig is associated with Fig 2. (PDF) [file pgen.1008515.s001.pdf]

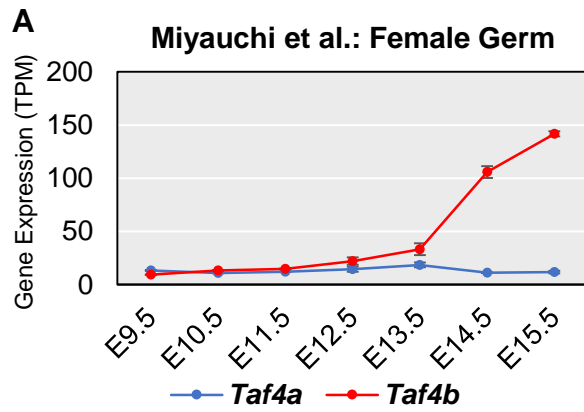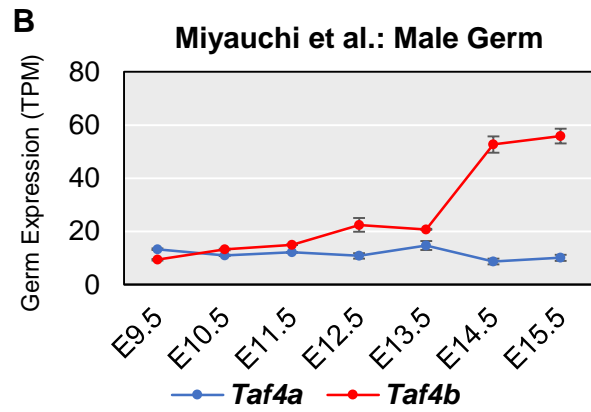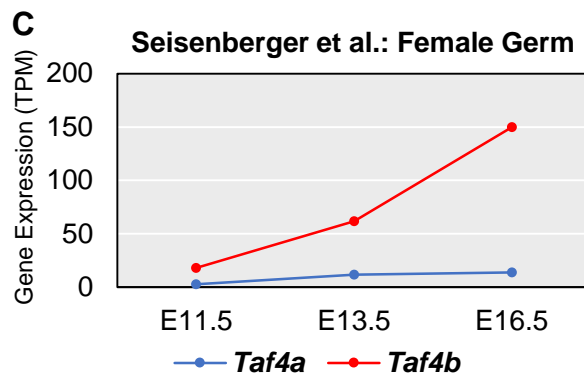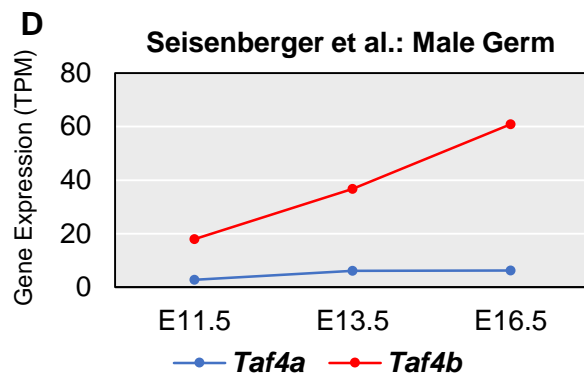

Supplement: S2 Fig — (A-B) RNA-seq from Miyauchi et al. [Supporting Information References 1] of cells sorted from Stella-EGFP E9.5-E13.5 and MVH-RFP E14.5-E15.5 female (A) and male (B) mice. For E9.5-E11.5, sexes were pooled together and the same data appear in both plots. (C-D) RNA-seq from Seisenberger et al. [Supporting Information References 2] of cells sorted from Oct4-EGFP female (C) and male (D) mice. For E11.5, sexes were pooled together, and the same data point appears in both plots. S2 Fig is associated with Fig 2. (PDF) [file pgen.1008515.s002.pdf]

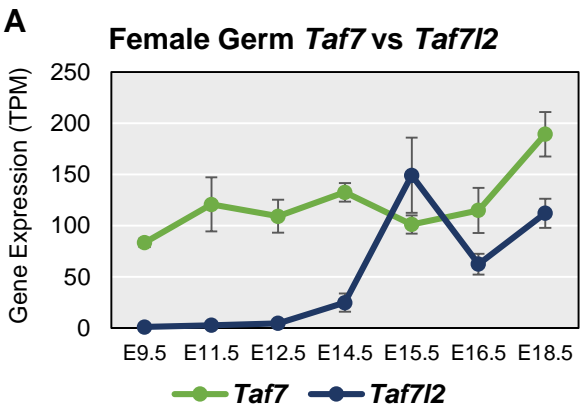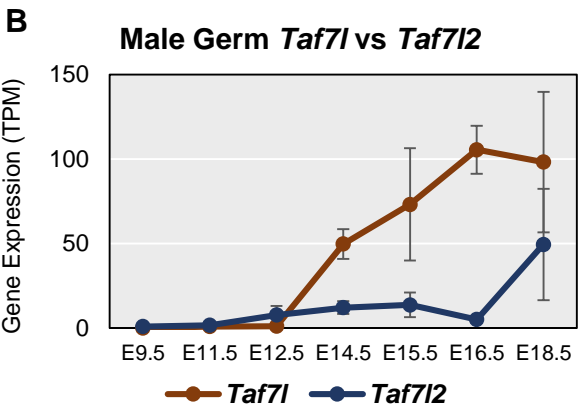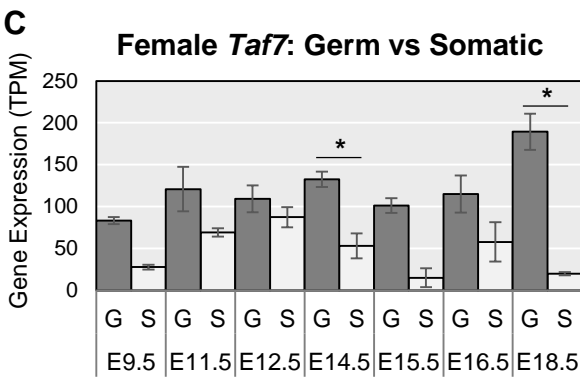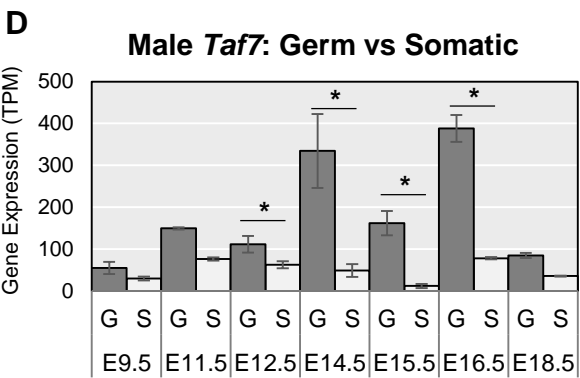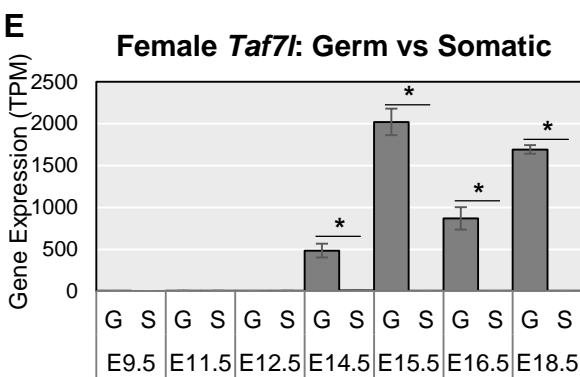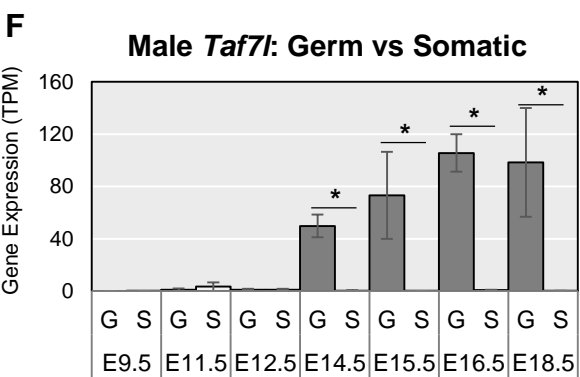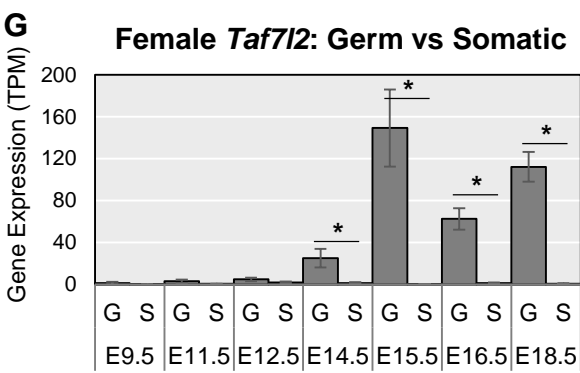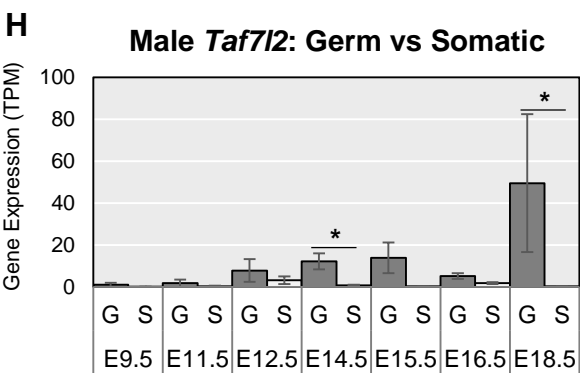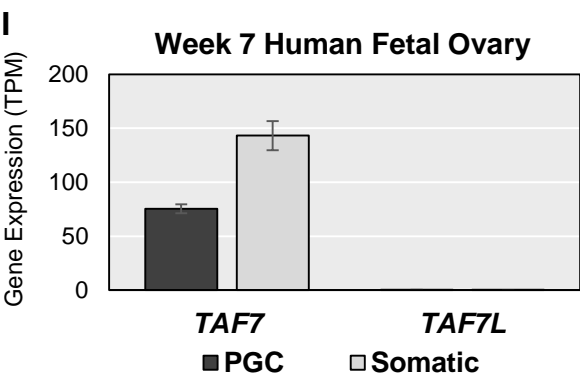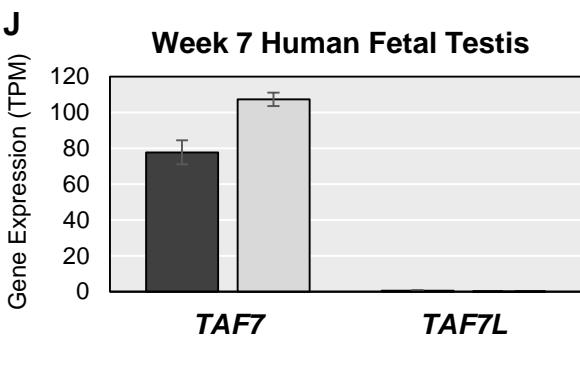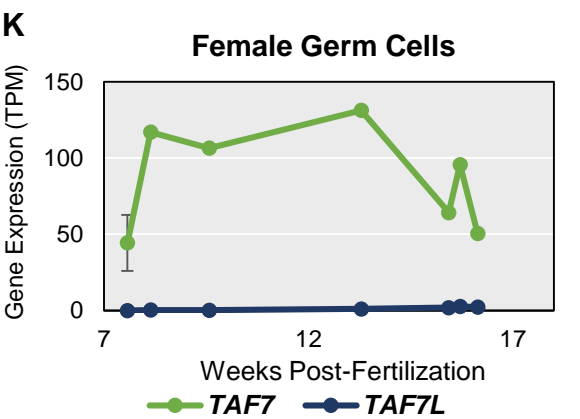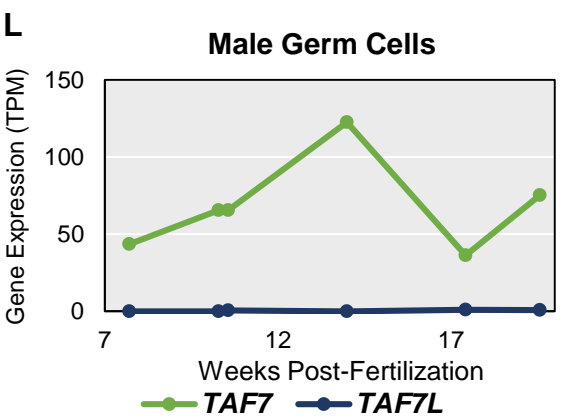

**M** Significantly Germ Cell-Enriched TFIIID Components

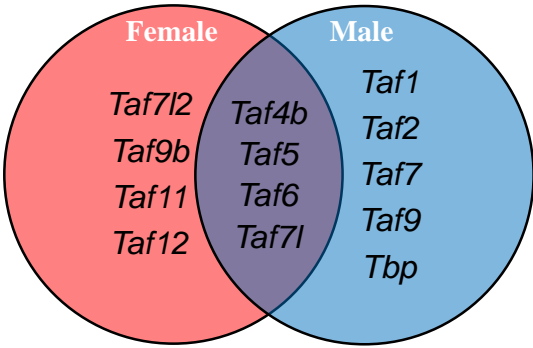

Supplement: S3 Fig — (A) Removal of Taf7l expression to more closely examine female germ cell expression of Taf7 and Taf7l2 over time. (B) Removal of Taf7 expression to more closely examine male germ cell expression of Taf7l and Taf7l2 over time. Expression of female (C, E, G) and male (D, F, H) mRNAs of Taf7, Taf7l, and Taf7l2 in germ cells (“G”) and somatic cells (“S”) from E9.5 to E18.5 (* = log2FC > |0.25|, p-adj. < 0.05). (I-J) TAF7 and TAF7L expression in human gonads indicate that TAF7L is barely detectable at 7 weeks post-fertilization. Taf7l2 does not have a human homolog. (K-L) From ~8 to ~16 weeks post-fertilization in females and to ~20 weeks post-fertilization in males, human TAF7L mRNA expression is low and unchanging while TAF7 expression is variable over time. This is dissimilar to the mouse TAF7L RNA-seq data. Error bars indicate ± SEM. (M) Venn diagram of TFIID subunits identified as significantly germ cell-enriched in the female and/or male germ cell time course data. S3 Fig is associated with Fig 2, Fig 3 and Fig 4. (PDF) [file pgen.1008515.s003.pdf]

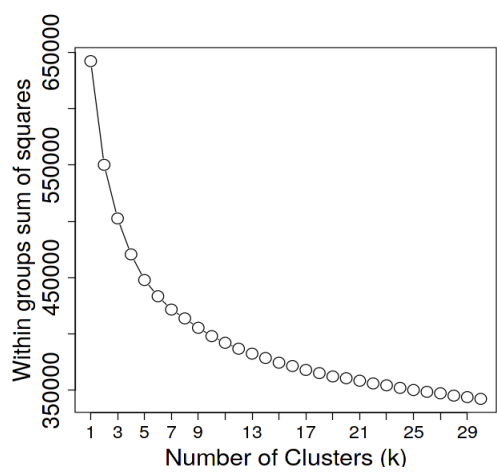

Supplement: S4 Fig — Nine clusters were chosen. S4 Fig is associated with Fig 5 and Table 1. (PDF) [file pgen.1008515.s004.pdf]

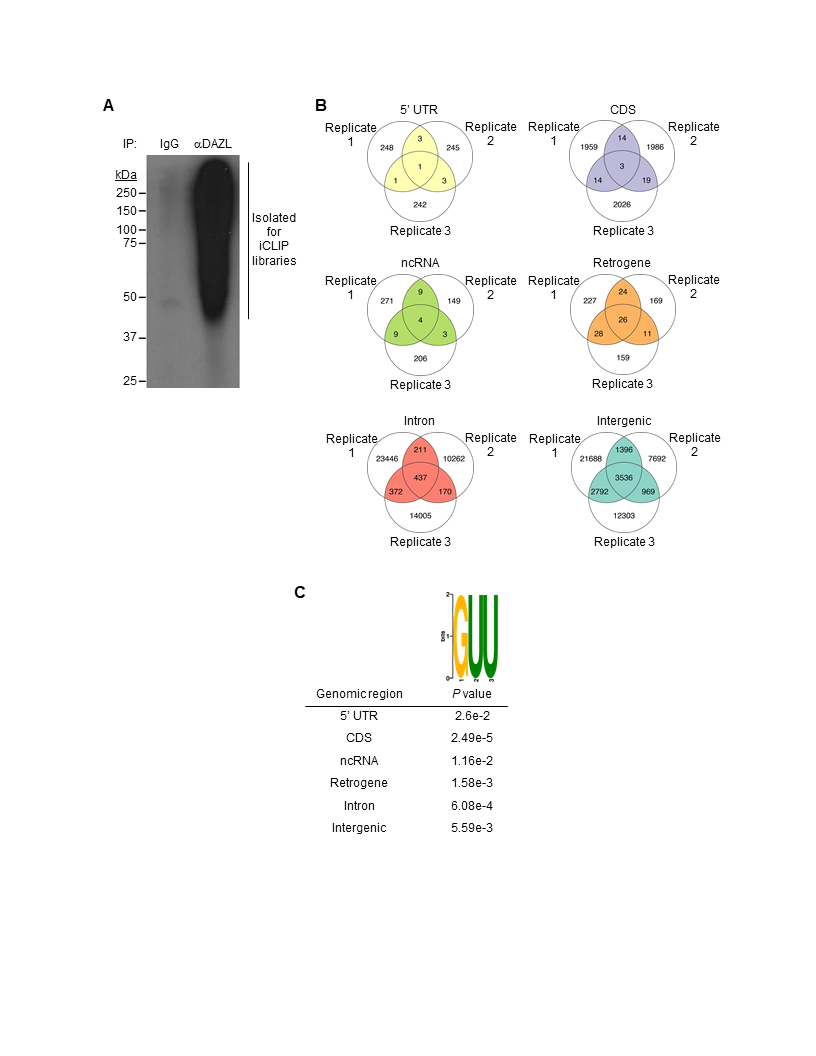

Supplement: S5 Fig — (A) Radioblot of DAZL:RNA complexes from postnatal testes synchronized for leptotene spermatocytes. DAZL:RNA complexes are larger than 37 kDa, the approximate molecular weight of DAZL. One of three biological replicates used to prepare iCLIP libraries reported here. (B) Venn diagram showing overlap of DAZL iCLIP peaks among 3 biological replicates in genomic regions other than the 3’ UTR. (C) Enrichment of DAZL’s GUU motif at replicated iCLIP peaks from genomic regions other than the 3’ UTR. AME from the MEME Suite was used to identify motif enrichment at crosslinked nucleotides in replicated peaks relative to shuffled control sequences. S5 Fig is associated with Fig 8. (TIF) [file pgen.1008515.s005.tif]
