# Supplementary material for: Dynamic and regulated TAF gene expression during mouse embryonic germ cell development
Source: PLoS Genet. 2020 Jan 8;16(1):e1008515. doi: 10.1371/journal.pgen.1008515 (PMC7010400; doi:10.1371/journal.pgen.1008515)

A

TFIID	# of Replicates with Stra8-FLAG Promoter Peak Called	# of Replicates with Input Peaks Called
Taf1	1	0
Taf2	0	0
Taf3	2	0
Taf4a	1	0
Taf4b	3	0
Taf5	0	0
Taf6	1	0
Taf7	0	0
Taf7l	3	0
Taf7l2	3	0
Taf8	0	0
Taf9	2	0
Taf9b	0	0
Taf10	1	0
Taf11	2	0
Taf12	1	0
Taf13	0	0
Tbp	0	0

B

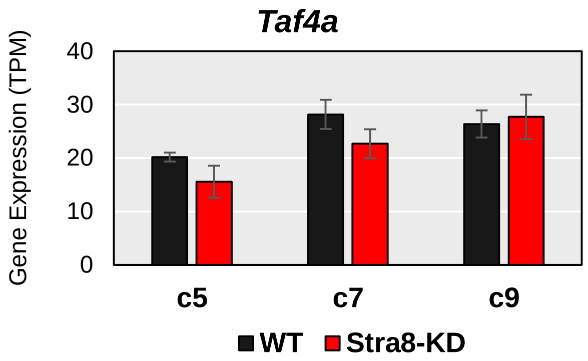

C

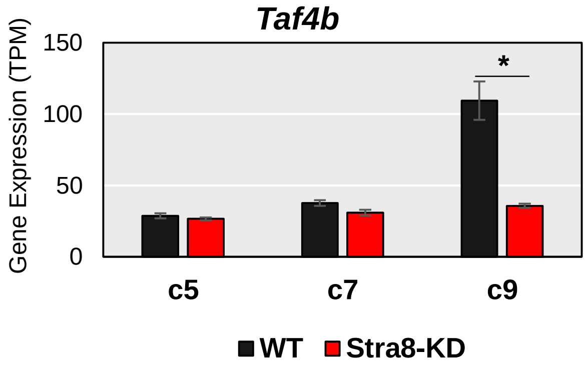

D

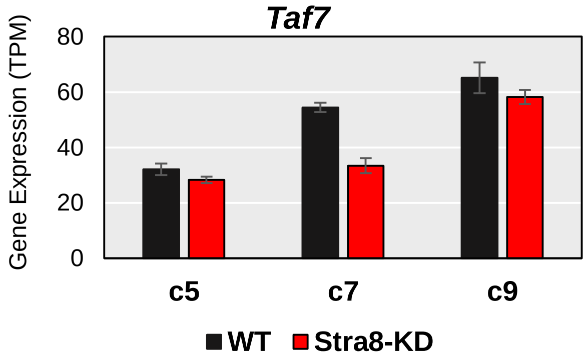

E

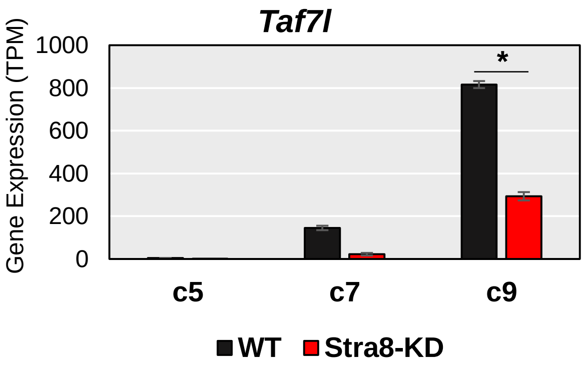

F

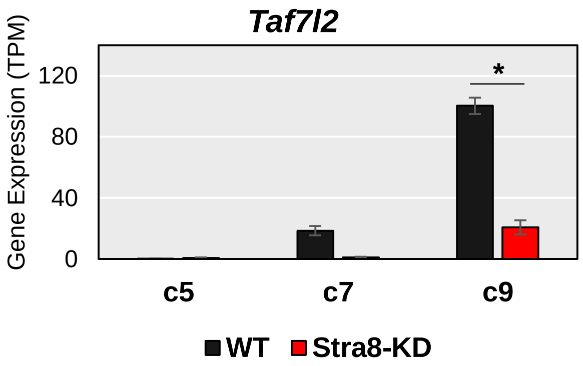

Supplement: S6 Fig — (A) STRA8-FLAG peaks called by MACS2 at the transcription start sites of TFIID components in comparison to the DNA input control. (B-F) mRNA expression levels in WT and Stra8-knockdown in vitro differentiated PGCLCs from reprocessed Miyauchi et al. RNA-seq using retinoic acid and BMP2. Expression of Taf4a (B) and Taf7 (D) do not see a strong induction at culture day 9 (c9) nor are differentially expressed in the Stra8-knockdown. Taf4b (C), Taf7l (E), and Taf7l2 (F) are highly expressed at c9 but fail to be induced in Stra8-knockdown cells (* = log2FC > |0.25|, p-adj. < 0.05). S6 Fig is associated with Fig 7. (PDF) [file pgen.1008515.s006.pdf]
